# Targeting DNA G-Quadruplexes with Helical Small Molecules

**DOI:** 10.1002/cbic.201402439

**Published:** 2014-09-26

**Authors:** Sebastian Müller, Katta Laxmi-Reddy, Prakrit V Jena, Benoit Baptiste, Zeyuan Dong, Frédéric Godde, Taekjip Ha, Raphaël Rodriguez, Shankar Balasubramanian, Ivan Huc

**Affiliations:** [a] Université de Bordeaux, CBMN, UMR 5248, Institut Européen de Chimie Biologie2 rue Escarpit, 33607 Pessac (France) and CNRS, CBMN, UMR 5248 (France); [b] Department of Chemistry, University of CambridgeLensfield Road, Cambridge CB2 1EW (UK); [c] Cambridge Institute, Cancer Research UK, Li Ka Shing CenterCambridge CB2 0RE (UK); [d] Department of Physics, Howard Hughes Medical Institute, University of Illinois at Urbana–ChampaignUrbana, IL 61801 (USA)

**Keywords:** DNA structures, foldamers, FRET, G-quadruplex, single-molecule fluorescence

## Abstract

We previously identified quinoline-based oligoamide helical foldamers and a trimeric macrocycle as selective ligands of DNA quadruplexes. Their helical structures might permit targeting of the backbone loops and grooves of G-quadruplexes instead of the G-tetrads. Given the vast array of morphologies G-quadruplex structures can adopt, this might be a way to achieve sequence selective binding. Here, we describe the design and synthesis of molecules based on macrocyclic and helically folded oligoamides. We tested their ability to interact with the human telomeric G-quadruplex and an array of promoter G-quadruplexes by using FRET melting assay and single-molecule FRET. Our results show that they constitute very potent ligands—comparable to the best so far reported. Their modes of interaction differ from those of traditional tetrad binders, thus opening avenues for the development of molecules specific for certain G-quadruplex conformations.

## Introduction

Chromatin is organized into distinct regions that are defined by their biochemical environment. Intricate protein networks are involved in maintaining chromatin structure and function, and DNA can adopt distinct secondary structures besides the standard B-DNA helix;[1] this adds to the structural repertoire controlling the functionality of chromatin. Certain G-rich sequences can adopt supramolecular structures called G-quadruplexes, which comprise tetrads of Hoogsteen hydrogen-bonded guanines that stack with π–π interactions. These structures are stabilized by monovalent cations such as Na^+^ and K^+^ in the central electron-rich channel. The conformations of the strands and loops connecting the tetrads vary, depending on the nucleic acid primary sequence and factors such as strand number, salt composition, and concentration.[2] Genome-wide analyses have revealed that putative G-quadruplex sequences are abundant in the human genome, with enrichment in promoter regions.[3] They are also found at telomeres, which contain the repetitive G-rich sequence (TTAGGG)_*n*_ that can fold into G-quadruplexes.[4] This suggests that these elements play important roles in controlling gene expression[5] and telomere maintenance. In a seminal paper, Zahler et al. demonstrated the formation of G-quadruplexes from telomeric sequences in vitro, thus rendering them resistant to extension by the reverse transcriptase telomerase.[6] In addition, G-rich RNA can also fold into G-quadruplexes, and these might be important for the control of gene expression (e.g., translation, unsilencing imprinted genes) and telomere maintenance. G-quadruplex-interacting molecules can stabilize RNA G-quadruplexes, and this can lead to down-regulation of translation, thus implicating them as regulatory elements.[7] RNA G-quadruplexes thus certainly constitute interesting structures with possible biological consequences.[8]

Recent evidence supports the formation of G-quadruplexes in vivo, including nucleic acid pull-down strategies,[9] in vivo labeling and genome-wide sequencing with small molecules,[10] and the use of specific antibodies. A recent report describing the visualization of RNA G-quadruplexes in the cytoplasm of human cells, and this might provide new avenues to study RNA G-quadruplexes and their effects on RNA secondary structure. This approach can be used to monitor the effects of small molecules on RNA G-quadruplexes in cells.[7c, 11] Indeed, small molecules have proven to be formidable tools to study G-quadruplexes. They can exert various effects on cells, including gene expression patterns,[5b] induction of telomere shortening[12] and uncapping,[13] and induction of DNA damage.[10, 13b] Whereas the DNA damage response can be activated through telomere uncapping,[14] genome-wide analysis of the DNA damage response marker γH2A.X upon treatment with the G-quadruplex interactor pyridostatin revealed hotspots dispersed over genomic DNA that contains clusters of putative G-quadruplex-forming sequences.[10] Given the possible involvement of G-quadruplexes in a plethora of biological processes, there is scope for the development of therapeutic agents based on G-quadruplex ligands, in order to interfere with these processes in pathological situations.[15]

Most of the molecules designed to interact with G-quadruplexes comprise an electron-poor flat aromatic surface surrounded by cationic charges,[16] in order to target the external tetrads of the nucleic acid structure,[13b, 17] thereby putting forward the notion that such small molecules should ideally be flat and aromatic in nature. However, an increasing number of ligands have been reported with alternative modes of interaction, for instance a chiral cyclic helicene,[18] distamycin A (and some of its derivatives), which was shown by NMR spectroscopy to be quadruplex groove binders,[19] the loop-binder DODC,[20] the groove binder Toxapy,[21] binol derivatives,[22] supramolecular complexes,[23] and synthetic G-quartets.[24] In order to exploit such interactions further, foldamers have emerged as an interesting class of compounds, as they are able to adopt well-defined structures stabilized by non-covalent interactions mimicking the structures of biopolymers.[25] Examples of foldamers that have been developed for biological applications such as G-quadruplex binding include peptidic nucleic acids (PNA), peptoids, and β, γ, δ, and ε peptides.[26] These molecules are small-to-medium sized, often resistant to proteolytic cleavage,[27] and show good cell permeability.[28]

**Scheme 1:**
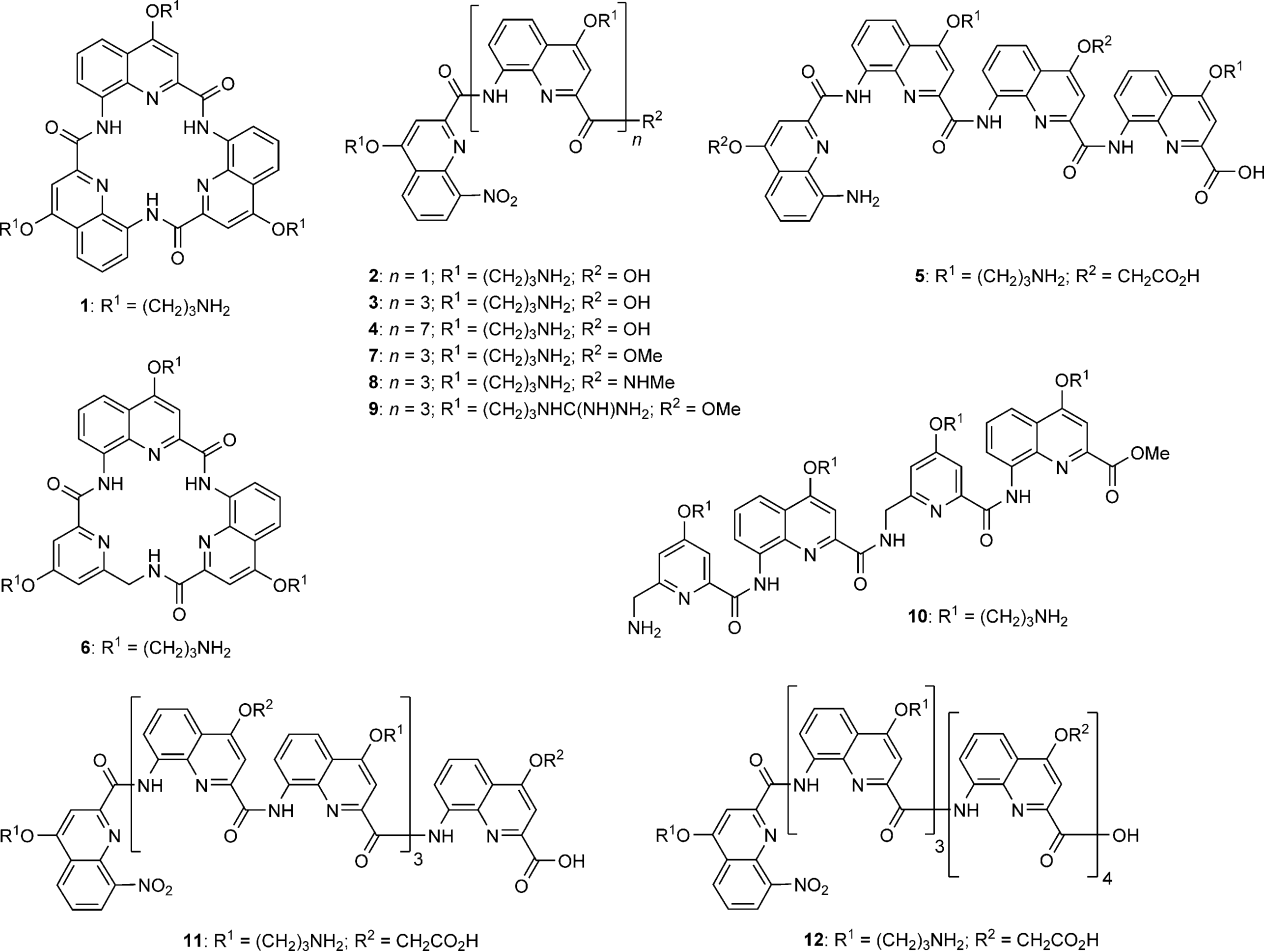
Macrocycles and foldamers synthesized for this study, derived from 8-amino-quinolinecarboxylic acids and 5-aminomethyl-pyridinecarboxylic acid. Some secondary amide structures are shown as *cis* conformers for clarity but exist as *trans* conformers in the folded helices.

Previously, we reported a quinoline-based macrocycle **1** and dimeric and tetrameric foldamers **2** and **3** (Scheme 1) as G-quadruplex-interacting molecules.[29] Macrocycle **1** was a very potent ligand and falls into the category of planar aromatic compounds with positive cationic side chains. In contrast, tetrameric foldamer **3** is helical, (i.e., non planar), yet it also displayed remarkable stabilization of the human telomeric G-quadruplex and the *c-kit* promoter G-quadruplex. These interactions were shown to depend on helix handedness, as CD experiments revealed that they resulted in a preferred helix sense for **3**. Subsequently, we dissected the interactions of aromatic oligoamide foldamers with nucleic acids by using directed DNA evolution against a helical cationic foldamer. We confirmed that G-quadruplexes stand as preferred targets and found that foldamers specifically interact with the backbones of G-quadruplexes (loops or grooves) as opposed to the top or bottom tetrads.[30] We also observed preference for DNA– quadruplex binding over RNA–quadruplex binding, and noted foldamer helix handedness and sequence dependence of the foldamer–quadruplex interaction.[30] Thus, macrocyclic and folded helical oligoamides selectively interact with G-quadruplexes[17c, 29,30]

Inspired by studies showing that derivatives of G-quadruplex ligands can be better than the lead structures[12,31] and that controlled folding of a small molecule can enhance G-quadruplex selectivity,[32] we endeavored to synthesize a new series of aromatic amide ligands: nine helical foldamers and a macrocycle. We evaluated their ability to stabilize the human telomeric G-quadruplex and a number of promoter G-quadruplexes by using FRET, and we investigated the interactions of two of the best foldamers further by single-molecule FRET. This method has the merit that it allows comparison to other well-established G-quadruplex ligands. We demonstrate that our new molecules interact specifically with G-quadruplex DNA (over duplex DNA) and compare favorably with the best G-quadruplex-stabilizing molecules reported so far.[13b, 29] Interestingly, we found that some of the synthesized helical foldamers showed better stabilization potential than macrocycles in this family.

## Results and Discussion

### Synthesis and design of small molecules

We previously reported the syntheses of macrocycle **1**, dimeric and tetrameric foldamers **2** and **3**,[29] and foldamers **4**[33] and **5**.[34] All are based on quinolinecarboxamide sequences. Macrocycle **1** and dimer **2** adopt a flat conformation,[35] whereas a helical conformation is adopted by tetramers **3** and **5 (**1.5 turns) and octamer **4** (over three turns). For the purpose of this study, we synthesized another macrocycle (**6**) to compare it to **1**. Macrocycle **6** has the same ring atom number as **1**, but one of the quinolines is replaced by an aminomethylpyridine derivative that was prepared using a previously described procedure.[34,36] The preparation of **1** involved the low-yielding direct cyclotrimerization of a monomer precursor, a scheme that allows the assembly of only three copies of the same monomer. In contrast, the preparation of macrocycle **6** involved the high-yielding cyclization of an isolated trimeric non-cyclic precursor. This new scheme is compatible with the sequential incorporation of different monomers. The yield of cyclization is enhanced by the high nucleophilicity of the benzylic amine in this step (see Supporting Information). The exact conformation of the resulting macrocycle was not investigated; it might adopt a planar conformation, but a distortion at the additional sp^3^ center might also favor nonplaner states, as observed in a related herringbone helical foldamer.[36]

Inspired by our previous report of the promising ability of the tetrameric foldamer **3**[29] to selectively stabilize G-quadruplex DNA, we varied its C-terminal group R^2^ and the nature of the side chains R^1^ to generate tetrameric foldamers **7**–**9**, and assessed the effects on G-quadruplex stabilization. The Boc-protected precursor of ester **7** was converted to the precursor of acid **3** by saponification, and also to the precursor of secondary amide **8** by using excess methylamine (see the Supporting Information). Side-chain deprotection of these precursors with trifluoroacetic acid (TFA) yielded **3**, **7**, and **8**. Compound **9** was synthesized by converting the propylammonium side chains of **7** to guanidinium by using 1*H*-pyrazol-1-carboxamide hydrochloride. The rationale was to create a side chain with a delocalized cationic charge, which might allow the formation of an arginine fork motif with the phosphate groups of the nucleic acid backbones of the loops and grooves of G-quadruplexes.[37]

Next, we synthesized tetramer **10** with alternating quinoline and methylaminopyridine building blocks by using simple coupling strategies (see the Supporting Information). This type of backbone can fold in a similar manner as homo-quinoline multimers, but with decreased stability. We did not synthesize homo-methylaminopyridine multimers as these species do not fold in organic solvents[36] or water.[34] In order to assess the effect of foldamer length on G-quadruplex recognition, we also synthesized the octameric foldamers **11** and **12** (see the Supporting Information), which carry cationic and anionic side chains potentially to introduce selectivity. These two molecules were made from quinoline building blocks but differ in the sequences of the side-chain substitutions. These synthetic procedures highlight how complex foldamers can be synthesized in a few steps in good yield.

### FRET melting analyses

We assessed the interactions of the molecules with G-quadruplex DNA by employing the FRET melting assay of Mergny and Maurizot.[38] This assay is widely established in the field to assess the potency of G-quadruplex ligands and is thus a good method to compare novel molecules with the plethora of reported ligands. It proved to be an efficient method to investigate our family of foldamers as G-quadruplex ligands in comparison with other studies. We focused on DNA quadruplex sequences and used the human telomeric G-quadruplex (H-telo)[6] and the sequences of the promoter quadruplexes of a selection of genes: *c-kit* (which contains the two G-quadruplex sequences c-kit1[39] and c-kit2[40]), *c-myc*,[5b, 41] *bcl2*,[42] and *k-ras*.[43] These sequences differ in composition, number of nucleotides in each loop, and they adopt different quadruplex conformations, thus potentially allowing differential recognition by small molecules that do not target the tetrads per se. As a control we used a sequence that forms duplex DNA in solution. We labeled each of these sequences with 6-carboxyfluorescein (FAM) and 6-carboxytetramethylrhodamine (TAMRA) at the 5′- and 3′-ends, respectively. Melting of G-quadruplexes results in an increase in the distance between the fluorophores, and this can be measured by changes in the FRET signal. We measured the changes in melting temperature (Δ*T*_m_) upon varying the concentration of added foldamer or macrocyle (Δ*T*_m_ in presence of 1 μm ligand in Table 1; concentrations for maximal stabilization in Table 2; full melting profiles in the Supporting Information).

**Table 1:** Δ*T*_m_ in 60 mm K^+^ at 1 μm compound in the FRET melting assays.

Ligand	Duplex[a]	H-Telo	c-kit1	c-kit2	c-myc	bcl2	k-ras
Δ*T*_m max_ [°C][b]	32.8±1.2	36.1±1.3	41.0±1.5	22.8±0.7	16.2±1.1	32.1±0.9	49.6±1.4
1	0.7	24.0	16.0	21.4	6.2	14.6	7.3
2	0.0	3.6	10.1	4.4	2.1	0.5	2.8
3	1.1	22.4	15.1	17.6	14.3	18.9	20.8
4	3.9	36.5	39.1	21.5	17.0	30.7	48.4
5	0.0	0.5	2.7	0.0	0.2	0.0	0.0
6	1.6	29.0	13.4	4.8	16.4	13.4	10.5
7	1.3	36.4	37.9	16.8	16.4	30.8	24.1
8	0.0	35.1	24.0	15.9	17.0	19.9	14.2
9	0.6	22.1	20.2	9.9	8.5	12.3	18.4
10	3.1	34.8	25.8	21.0	16.5	27.7	26.8
11	0.0	0.0	0.0	0.0	0.0	0.0	0.2
12	0.0	2.8	6.1	2.8	1.4	0.2	1.8

[a] Duplex formed by two complementary strands linked by a hexa(ethylene glycol) loop.

[b] This value corresponds to 95.5 °C−*T*_m_ [°C] for quadruplex alone, as 95.5 °C was the maximum measurable temperature.

**Table 2:** Concentration [μm] required for maximal stabilization in the FRET-melting assays.

Ligand	Duplex[a]	H-Telo	c-kit1	c-kit2	c-myc	bcl2	k-ras
1	6.3	4.1	2.9	1.6	8.1	4.6	6.5
2	>10	>10	>10	>10	>10	>10	>10
3	9.7	4.4	4.3	2.9	2.1	4.7	9.0
4	6.10	0.36	0.34	0.8	0.34	0.56	0.69
5	>10	>10	>10	>10	>10	>10	>10
6	>10	1.41	6.01	5.3	1.32	2.29	>10
7	4.70	0.92	1.94	2.0	1.07	1.23	6.83
8	5.23	1.25	3.69	3.1	0.85	3.09	6.42
9	>10	2.4	3.8	5.4	2.2	2.1	4.2
10	6.77	1.18	4.39	1.3	0.77	1.58	5.57
11	>10	>10	>10	>10	>10	>10	>10
12	>10	>10	>10	>10	>10	>10	>10

[a] Duplex formed by two complementary strands linked by a hexa(ethylene glycol) loop.

Because of differences in sequence and structure, the various G-quadruplexes exhibited different *T*_m_ values. The maximal Δ*T*_m_ values also differed substantially (from 16.2 (*c-myc*) to 49.6 °C (*k-ras*); Table 1). Changes in melting temperature (below) provide information about the potential of the ligands to interact with G-quadruplexes. Nevertheless, the differences mentioned above, along with the fact that G-quadruplexes can change conformation upon binding one ligand but not another, call for great caution when drawing comparisons. In addition, interactions between ligands and quadruplexes that do not lead to quadruplex stabilization were not examined in these assays.

As a complement to Δ*T*_m_ values, we attempted to measure dissociation constants (*K*_d_) by using surface plasmon resonance (SPR). However, SPR measurements with DNA sequences attached to the chip failed because of non-specific interactions between the foldamers and the substrate of the chip. Better results might be obtained by attaching the foldamers to the chip.[30] But slow dissociation kinetics, possible quadruplex aggregation, and conformational changes complicated the measurements, which could not be fitted to a simple 1:1 binding model. Nevertheless, it can reasonably be inferred from the Δ*T*_m_ values that the lowest dissociation constants are in the low or sub-micromolar range. Accurate determination of binding constants would be possible by using specific techniques that are beyond the scope of this study.[44] It should also be kept in mind that neither *K*_d_ nor Δ*T*_m_ values provide sufficient information to ascertain quadruplex binding in cells and the triggering of a biological response.

The results show that all fully cationic oligomers, whether cyclic (**1**, **6**), flat and non cyclic (**2**), helical (**3**, **4**, **7**–**10**), long (**4**), or bearing ammonium or guanidinium (**9**) side chains, showed good to high stabilization with DNA quadruplexes and selectivity against duplex DNA. In contrast, the presence of anionic residues, either alternating with cationic residues (**5**, **11**) or clustered at one end of a sequence (**12**), was strongly detrimental to DNA binding. Earlier studies have shown that the high conformational stability of helices does not depend on monomer sequence.[34] Thus, the different behaviors cannot be assigned to different foldamer conformations resulting from a change in sequence. Instead, electrostatic repulsions between foldamer and DNA negative charges are probably responsible for this dramatic effect. It is worth noting that octamer **12** contains a tetrameric cationic N-terminal segment, which is a good ligand when tested independently. Nevertheless, this compound showed minimal G-quadruplex stabilization; the neighboring negative charges did not allow strong binding of the cationic segment.

Both macrocycles **1** and **6** strongly stabilized G-quadruplexes and had minimal effects with duplex DNA, thus confirming earlier results on the stabilization of H-telo by **1**.[29] The sp^3^ center of **6** appeared not to be an impediment. Data for the two compounds are overall comparable, but some notable differences are worth pointing out. Macrocycle **1** displayed a remarkable stabilization potential for c-kit2 whereas **6** was limited in this respect. Conversely, **6** fared much better than **1** and seemed to behave like helical oligomers concerning the stabilization of c-myc. This might be related to the fact that c-myc adopts an unusual propeller-type parallel-stranded conformation.[45]

A comparison of flat dimer **2**, helical tetramer **3**, and helical octamer **4**, revealed a major effect of length. Compound **2** exhibited weak stabilization for all G-quadruplex targets; **3** displayed very good stabilization (matching or surpassing **1** in most cases); strikingly, octamer **4** stabilized all G-quadruplex targets (maximal Δ*T*_m_ at 1 μm compound; Table 1). The concentration required to stabilize these targets by **4** was 0.36–0.80 μm (Table 2), whereas duplex stabilization was negligible. Thus this octameric foldamer is by far the most potent G-quadruplex stabilizing ligand in the series, and is comparable to the most-potent reported small molecules.[13b]

Changing the C-terminal negatively charged carboxylate function of **3** to a neutral ester (**7**) or methyl-amide (**8**) significantly enhanced G-quadruplex stabilization in almost all cases, thus confirming the detrimental effect of negative charges in side chains. Interestingly, the minor structural difference between **7** and **8** nevertheless results in some substantial differences in Δ*T*_m_ (c-kit1, Bcl2, and k-ras entries in Table 1). The ester appears to be more efficient than the methyl-amide.

The introduction of guanidinium side chains on foldamer **9** reduced G-quadruplex targeting efficiency, despite their ability to form salt bridges with phosphate ions enhanced by bidentate hydrogen bonding. However, the DNA melting profiles in the presence of **9** (see Supporting Information) show that this foldamer exhibited formidable selectivity for G-quadruplexes over duplex DNA, even though maximal stabilization occurred above 1 μm. In this respect, **9** is more selective than all the other foldamers and macrocycles of this family.

Finally, tetramer **10** showed G-quadruplex stabilization properties similar to those of tetramer **7**. Both are methyl esters and bear four ammonium side chains, but **10** is much more flexible and not expected to fold well because of its aminomethyl-pyridine units.[34] Compared to quinoline rings, these units have reduced surface area for aromatic stacking. Compound **10** also has an N-terminal ammonium function, but these features have weak or compensating effects.

Altogether, the FRET melting results demonstrate that we improved on the G-quadruplex stabilization potential of the lead compounds **1** and **3**. We also showed the selectivity potential of helical oligoamides with various G-quadruplex targets. This shows that these foldamers constitute a very potent family of G-quadruplex ligands.

### Single-molecule FRET analyses

**Figure 1:**
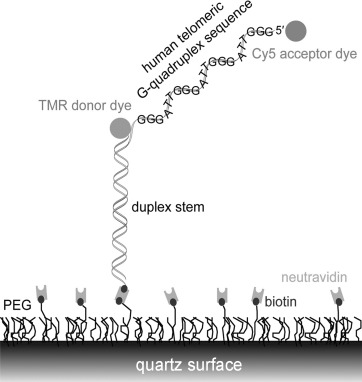
Single-molecule FRET.

To further dissect the conformations and dynamics of G-quadruplex DNA with foldamers and to get an insight into the binding mode, we investigated the interactions of **3** and **4** with the human telomeric G-quadruplex by single-molecule FRET. We tethered biotinylated DNA to a PEG-passivated quartz surface by using the specific biotin–neutravidin interaction (Figure 1).

**Figure 2:**
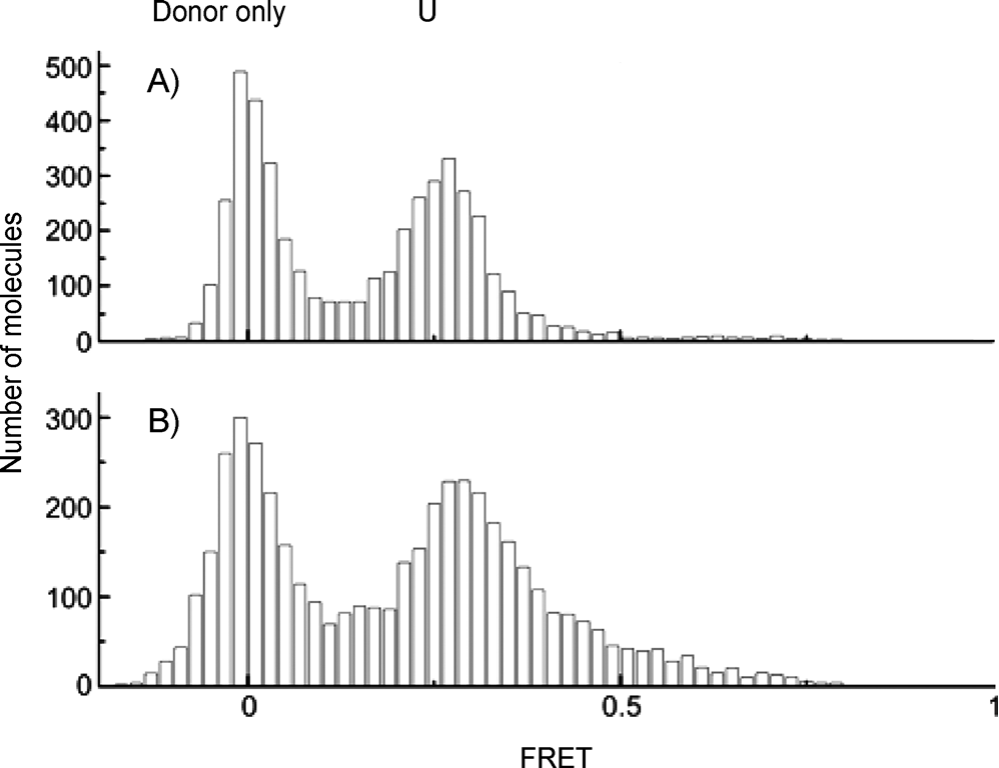
FRET histogram of human telomeric quadruplex: A) unfolded state in the absence of K^+^, and B) in the absence of K^+^ but upon addition of 100 nm tetramer 3.

This technique allows the monitoring of three different conformational states of H-telo: unfolded **U**, and resolvable folded conformations **F1** and **F2** (FRET efficiency *E*=0.43, 0.63 and 0.80, respectively). We reported previously that macrocycle **1** binds tightly to an unfolded telomeric strand, even in the absence of K^+^, and selectively stabilizes conformation **F1** over the naturally favored conformation **F2** of the human telomeric G-quadruplex.[17c] Unlike macrocycle **1**, the tetrameric foldamer **3** was unable to fold the human telomeric sequence into a G-quadruplex in the absence of K^+^ (Figure 2). Similarly, the octameric foldamer **4** did not induce folding of the sequence into a G-quadruplex (data not shown).

**Figure 3:**
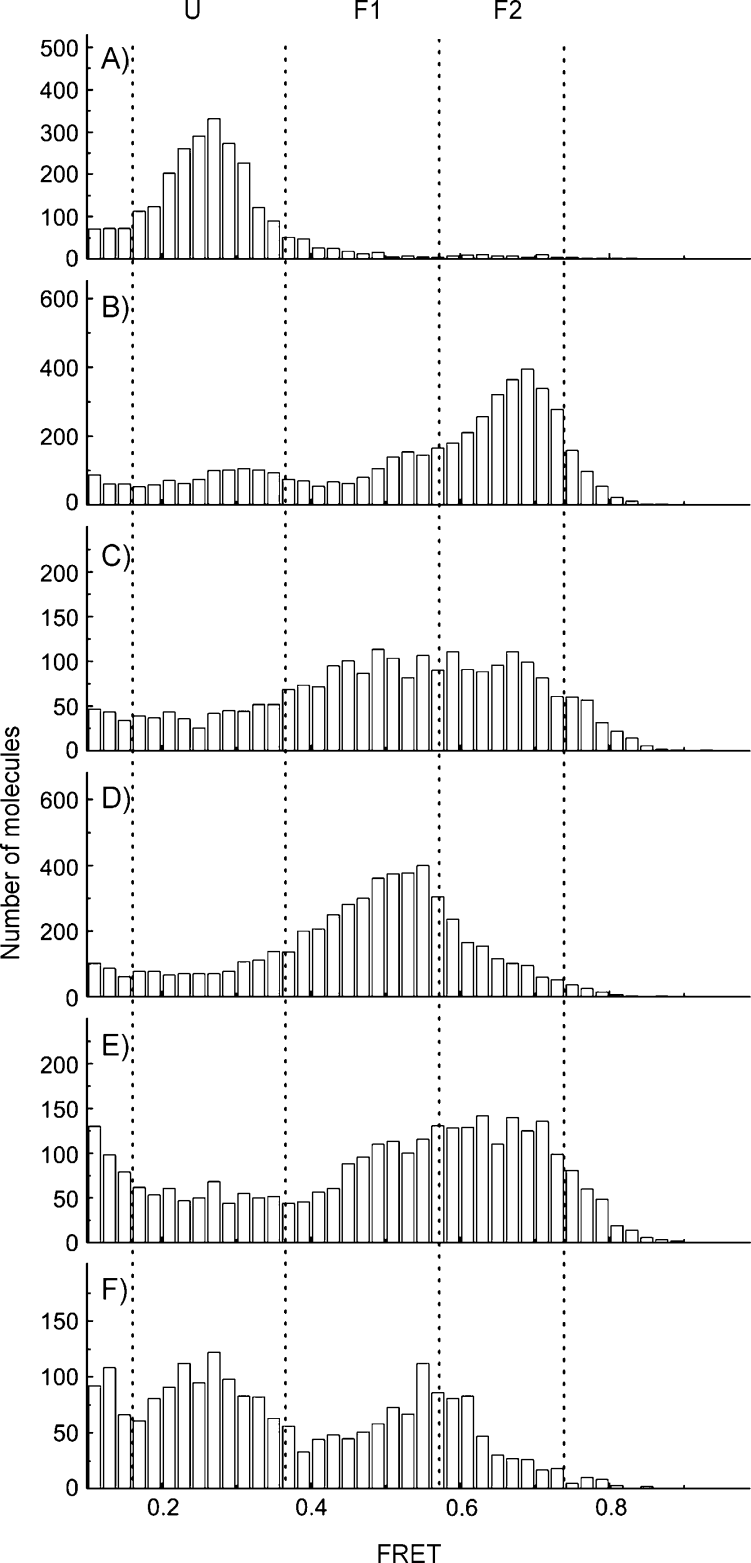
FRET histogram of H-Telo: A) unfolded state in the absence of K^+^, B) folded in F1 and F2 states upon addition of 10 mm K^+^, upon addition of tetramer 3 after C) 2 min and D) 20 min, E) upon addition of octamer 4 after 20 min, F) 30 min after removing free K^+^ and 4.

Addition of 10 mm K^+^ to the single-stranded human telomeric sequence led to the coexistence of two folded conformations (**F1** and predominantly **F2**; Figure 3 A and B). Subsequent addition of 100 nm **3** induced a rapid increase in the proportion of **F1** (Figure 3 **C**). To a lesser extent, **4** behaved in the same way (data not shown). After 20 min equilibrium was reached for both foldamers (Figure 3 D and E). Strikingly, unlike as previously observed for **1**, the folded state **F2** was not completely shifted to **F1** by **3** (Figure 3 D), and this was even more pronounced for **4** (Figure 3 E). When free **4** and K^+^ were removed (Figure 3 F), a mixed population of **F1** and **F2** remained, thus suggesting a strong stabilizing effect of still-associated foldamer **4** with the H-Telo G-quadruplex. Together, these data suggest a remarkably different mode of interaction between the macrocycle and the helical foldamers tested, and highlight a change in the dynamics of interaction between the tetrameric and octameric foldamer with the human telomeric G-quadruplex. This is in agreement with the shape of the molecules, which allow (**1**) or do not allow (**4**) direct stacking on top of the G-tetrads. The different behaviors of the helices and of the macrocycle is also in agreement with earlier circular dichroism data that suggested that **1** stabilizes the anti-parallel conformation of H-Telo,[29] and that **4** stabilizes the parallel conformation of all G-quadruplex aptamer sequences to which it was exposed.[30]

## Conclusions

The facile synthesis of a variety of foldamers based on helical oligoamides enabled us to generate a library of small molecules in a few simple synthetic steps. Most of these molecules showed very good stabilization for a variety of G-quadruplexes, as tested by FRET melting experiments. We have established helical oligoamides as a potent class of G-quadruplex-interacting molecules and showed that their modes of interaction differ from our previously reported macrocycle, which presumably interacts with the tetrads of the target nucleic acid structure. The helical foldamers thus possibly interact with the backbone loops and grooves G-quadruplexes.[17c, 30] The nature of the side chains and foldamer length play important roles in the stabilization ability of these foldamers with G-quadruplex nucleic acids. We found that the octameric foldamer **4** was even more potent than macrocycle **1**, thus placing it in the range of the best molecules reported so far. Given the relatively easy synthetic procedure and the modular nature, this family of ligands can easily be expanded further to generate even more potent molecules. As their modes of interaction with G-quadruplex DNA differ from those of traditional tetrad binders, this opens new avenues for the further development of molecules specific for certain G-quadruplex conformations. Thus, a detailed NMR spectroscopy and crystallographic structural investigation of a foldamer–quadruplex is currently in progress. Preliminary results with a co-crystal of **3** and the model DNA sequence G_4_T_4_G_4_ showed no contact between foldamer and G-tetrad.

It should be noted that foldamer/G-quadruplex adducts might trigger a different signaling response in a cellular environment, as compared to traditional tetrad binders, thereby providing a tool to shed light on G-quadruplex location and function in the genome. In addition, with the advent of the development of G-quadruplex-interacting small molecules as therapeutic agents,[15] and their good cell penetration ability,[28] these small molecules can potentially be used in biological assays and exploited for therapeutic development.

## Experimental Section

**FRET melting experiments:** Oligonucleotide stock solutions (100 μm in MilliQ water) were diluted in potassium cacodylate (60 mm, pH 7.4); FRET experiments were carried out with 200 nm oligonucleotide. The seven DNA oligonucleotides were dual fluorescently labeled. K-ras is a dual-labeled 32-mer oligonucleotide quadruplex from the promoter region of human *K-ras* (5′-FAM-AGG GCG GTG TGG GAA GAG GGA AGA GGG GGA GG-TAMRA-3′). C-kit1 was a dual-labeled 21-mer oligonucleotide representing one of the quadruplex-forming regions in the promoter of the human *c-kit* oncogene (5′-FAM-GGG AGG GCG CTG GGA GGA GGG-TAMRA-3′). C-kit2 was a similar dual-labeled 20-mer oligonucleotide (5′-FAM-GGG CGG GCG CGA GGG AGG GG-TAMRA-3′). H-Telo was a dual-labeled 21-mer oligonucleotide, the minimum human telomeric G-overhang sequence required to fold into an intramolecular quadruplex (5′-FAM-GGG TTA GGG TTA GGG TTA GGG-TAMRA-3′). C-myc was a dual-labeled 22-mer oligonucleotide comprising one of the quadruplex-forming regions in the promoter of the human *c-myc* oncogene (5′-FAM-TGA GGG TGG GTA GGG TGG GTA A-TAMRA-3′). Bcl2 was a dual-labeled 27-mer oligonucleotide comprising the quadruplex-forming region in the promoter of human *bcl2.* (5′-FAM-CGG GCG CGG GAG GAA GGG GGC GGG AGC-TAMRA-3′). ds-DNA was a dual-labeled self-complementary 20-mer oligonucleotide with a central polyethylene glycol linker able to fold into a hairpin (5′-FAM-TAT AGC TAT A HEG TAT AGC TAT A-TAMRA-3′; HEG: hexa(ethylene glycol)). The donor fluorophore was FAM, and the acceptor fluorophore was TAMRA. Dual-labeled DNA (400 nm) was annealed by heating at 94 °C for 10 min followed by cooling to room temperature (0.1 K min^−1^). 96-Well plates were prepared by addition of annealed DNA (50 μL) to each well, followed by the respective molecule (50 μL) at the required concentration. Measurements were made in triplicate with a LightCycler 480 (excitation 483 nm, detection 533 nm; Roche). Analysis of the data was carried out with OriginPro 7.5 software (OriginLab, Northampton, MA).

**Single-molecule FRET:** Single-molecule FRET studies were carried out as described previously.[17c] In brief, prism-type total internal reflection fluorescence (TIRF) microscopy was performed (532 nm laser excitation) to detect the conformation changes of surface-immobilized human telomeric DNA molecules labeled with a Cy5/TMR FRET pair. The detected intensity of TMR and Cy5 from each individual H-Telo molecule was used to derive FRET efficiency, and combined to obtain FRET histograms. Other experimental parameters were as in our previous single-molecule FRET study on H-Telo.
