# Supplementary material for: Targeting DNA G-Quadruplexes with Helical Small Molecules
Source: Chembiochem. 2014 Sep 26;15(17):2563–70. doi: 10.1002/cbic.201402439 (PMC4284101; doi:10.1002/cbic.201402439)

CHEMBIOCHEM

Supporting Information

© Copyright Wiley-VCH Verlag GmbH & Co. KGaA, 69451 Weinheim, 2014

Targeting DNA G-Quadruplexes with Helical Small Molecules

Sebastian Müller,[b, c] Katta Laxmi-Reddy,[a] Prakrit V. Jena,[d] Benoit Baptiste,[a] Zeyuan Dong,[a] Frédéric Godde,[a] Taekjip Ha,[d] Raphaël Rodriguez,[b, c] Shankar Balasubramanian,[b, c] and Ivan Huc*[a]

cbic_201402439_sm_miscellaneous_information.pdf

Supporting Information

Targeting DNA G-Quadruplexes with Helical Small Molecules

Sebastian Müller, Katta Laxmi-Reddy, Prakrit V. Jena, Benoît Baptiste, Zeyuan Dong, Frédéric Godde, Taekjip Ha, Raphaël Rodriguez, Shankar Balasubramanian, Ivan Huc

Table of contents

1. Synthetic Schemes Page S2
2. Materials and Methods Page S4
   2.1. FRET measurements Page S4
   2.2. Synthetic procedures Page S6
3. References Page S15
4. NMR spectra and chromatograms Page S16

1. Synthetic Schemes

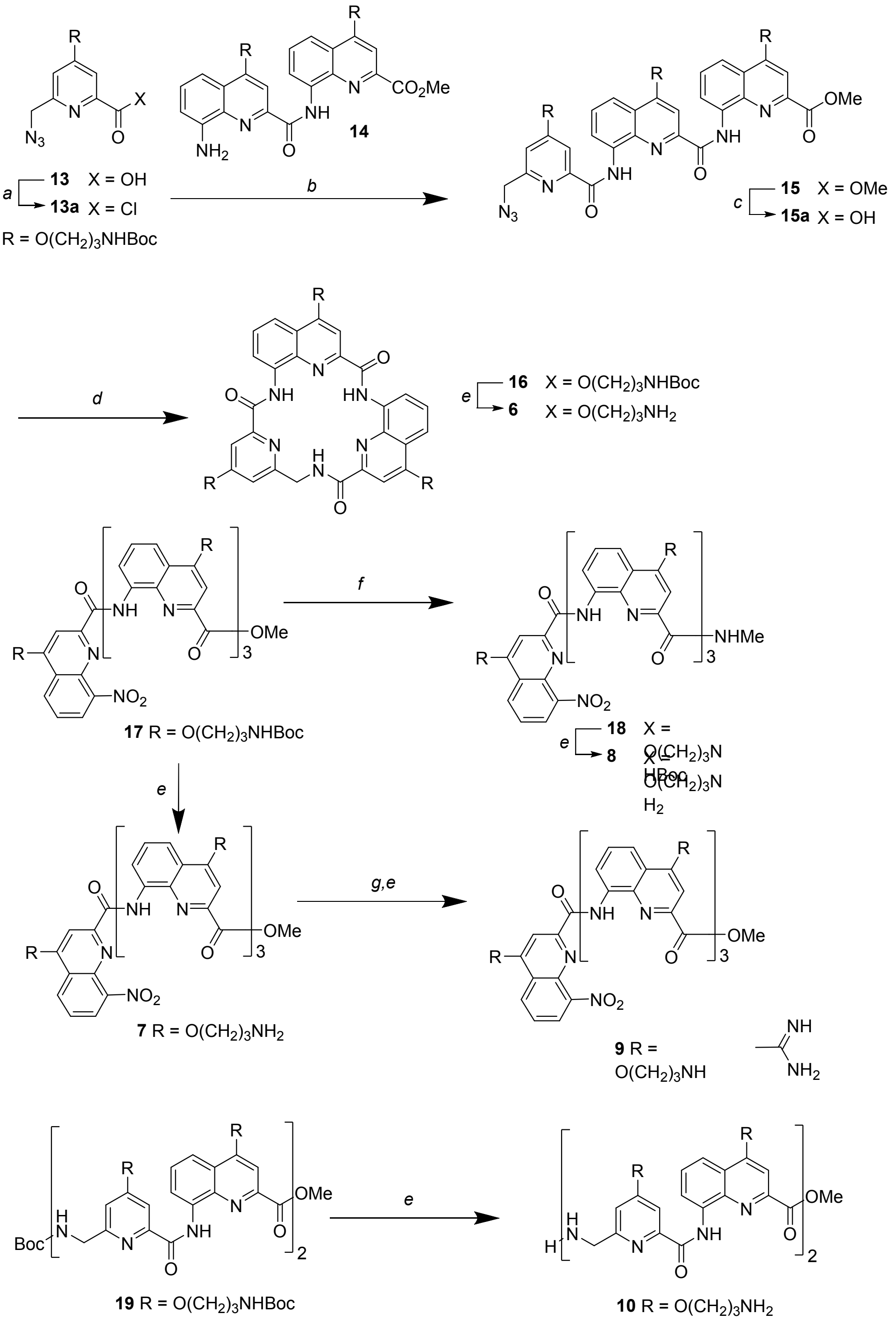

Scheme S1. Conditions: *a* 1-chloro-*N,N*-2-trimethylpropenylamine, CH_2Cl_2, 25°C, quantitative; *b* DIPEA, CH_2Cl_2, 25°C, 90%; *c* NaOH, THF/MeOH, 25°C, quantitative; *d* (i) EDCI, pentafluorophenol, CH_2Cl_2, 0°C then 25°C, (ii) H_2-Pd/C, THF, 25°C, (iii) DMAP, toluene, 100°C, 70% for three steps; *e*

TFA, CH_2Cl_2, 25°C, quantitative; *f* $MeNH_2$, THF/MeOH, 25°C, 80%; *g* 1-H-pyrazol-1-carboxamidine hydrochloride, Et_3N, DMF, 60%.

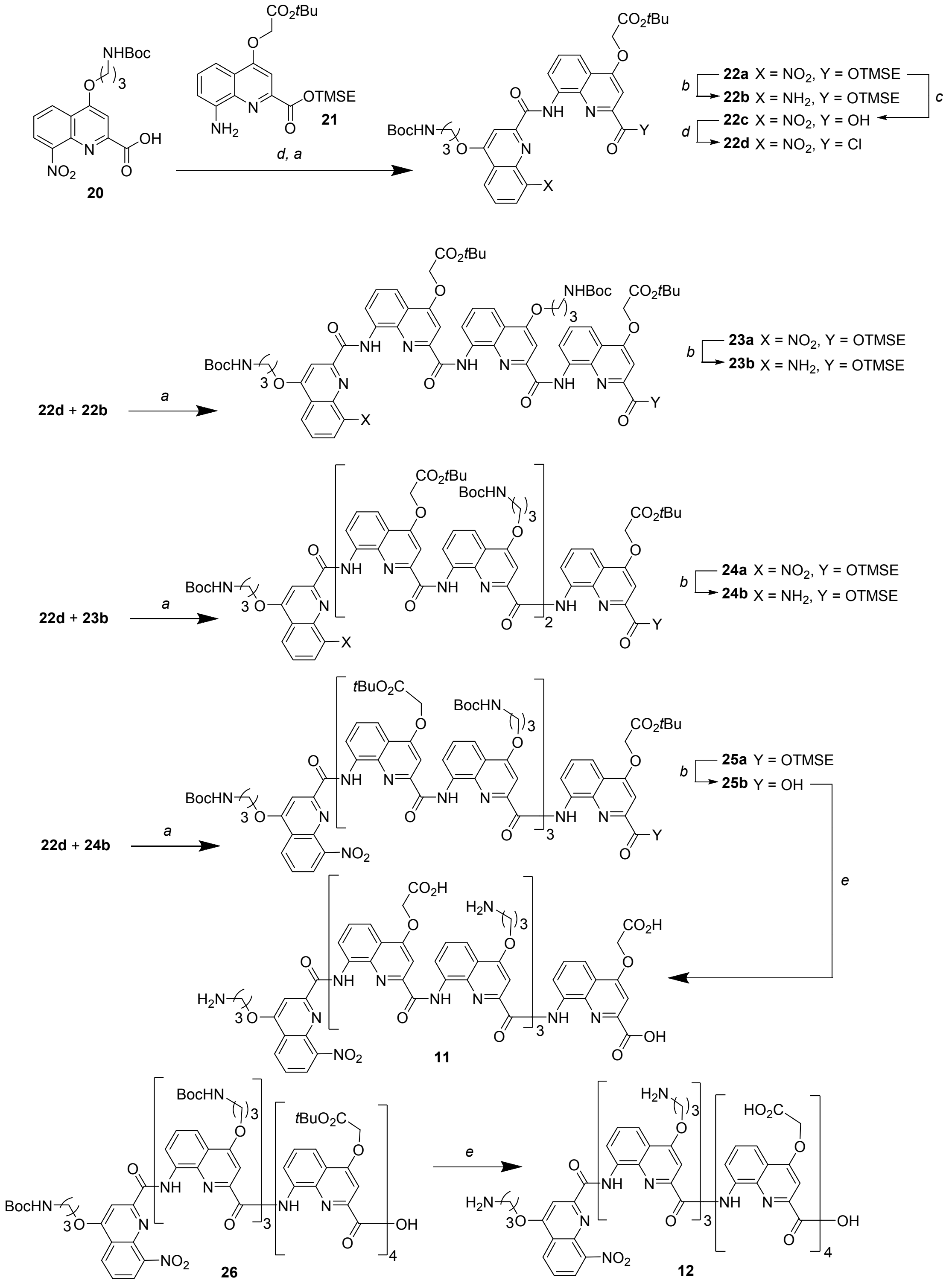

Scheme S1. Conditions: *a* DIPEA, CH_2Cl_2, 25°C, 80-85%; *b* H_2-Pd/C, EtOAc, 25°C, quantitative; *c* TBAF, THF, 25°C, 95%; *d* 1-chloro-*N*,*N*-2-trimethylpropenylamine, CH_2Cl_2, 25°C, quantitative; *e* TFA, CH_2Cl_2, 25°C, quantitative

2. Materials and Methods

2.1. FRET measurements

FRET-melting curves for compounds **1-12** against the various nucleic acid targets.

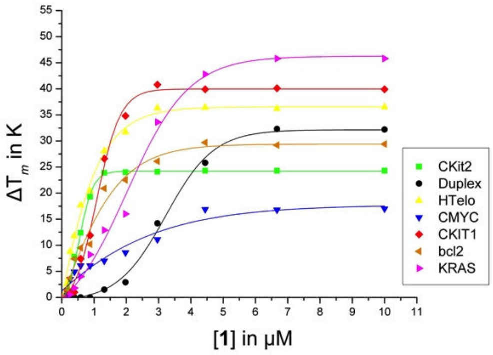

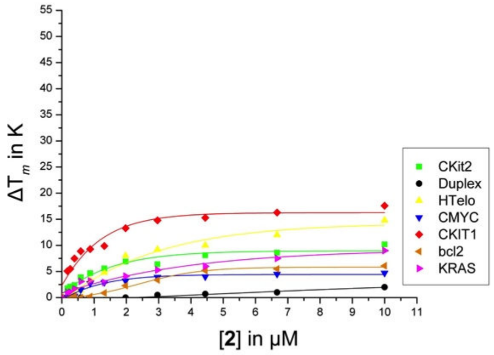

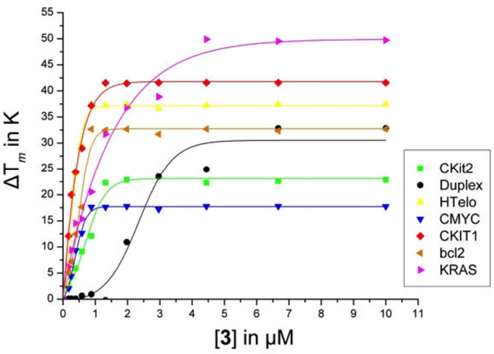

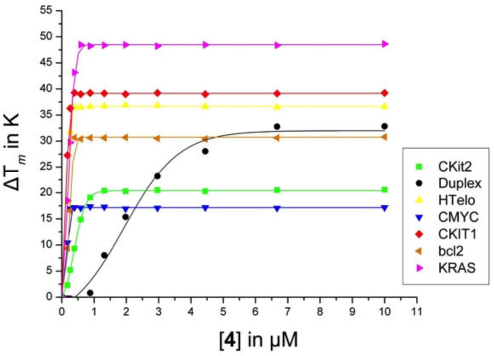

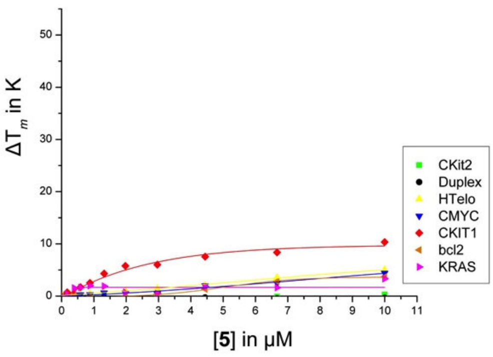

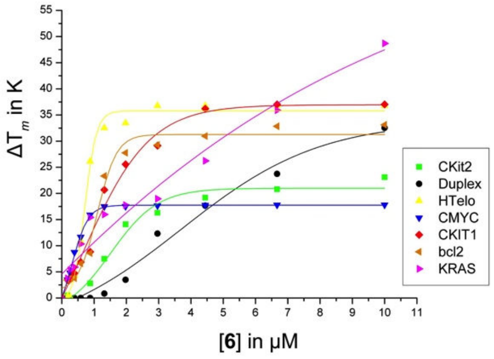

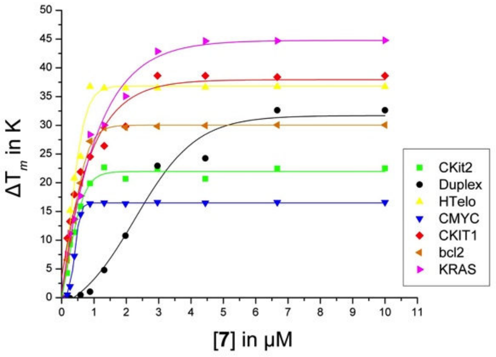
ΔT_m in K
CKit2
Duplex
HTelo
CMYC
CKIT1
bcl2
KRAS
[7] in µM

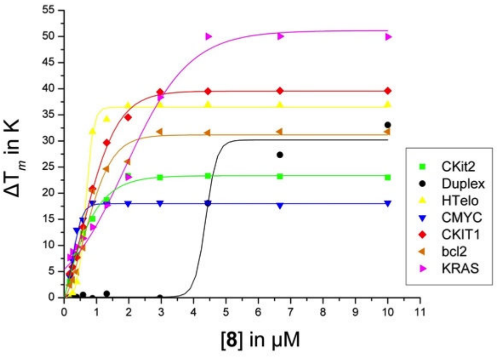
ΔT_m in K
CKit2
Duplex
HTelo
CMYC
CKIT1
bcl2
KRAS
[8] in µM

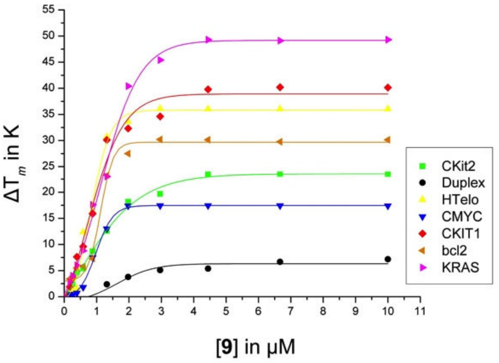
ΔT_m in K
CKit2
Duplex
HTelo
CMYC
CKIT1
bcl2
KRAS
[9] in µM

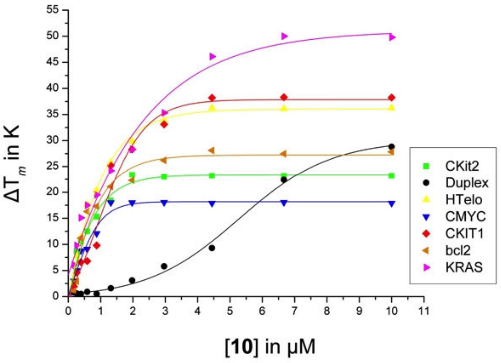
ΔT_m in K
CKit2
Duplex
HTelo
CMYC
CKIT1
bcl2
KRAS
[10] in µM

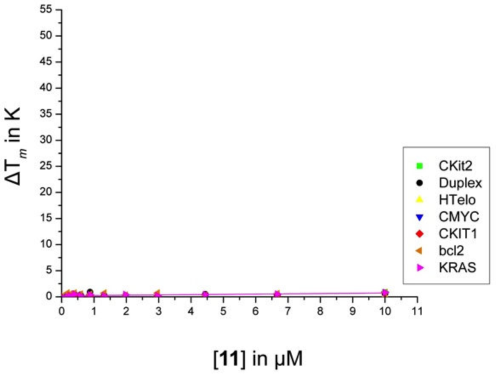
ΔT_m in K
CKit2
Duplex
HTelo
CMYC
CKIT1
bcl2
KRAS
[11] in µM

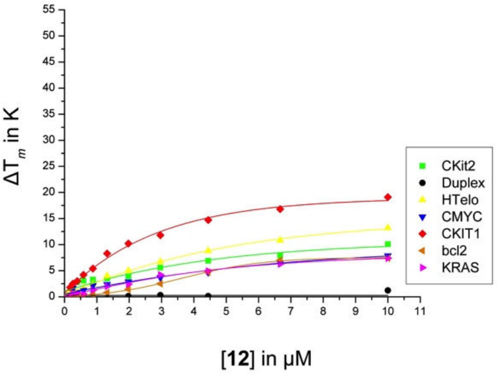
ΔT_m in K
CKit2
Duplex
HTelo
CMYC
CKIT1
bcl2
KRAS
[12] in µM

2.2. Synthetic procedures

General. Compounds **4**, **14**, **17**, and **20** were prepared as described in reference 1. Compounds **1**, **2**, and **3** were prepared as described in reference 2. Compounds **5, 13**, **19** and **20** were prepared as described in reference 3. Compounds **21** and **26** were prepared as described in reference 4. Unless otherwise noted, materials were obtained from commercial suppliers and used without further purification. CH_2Cl_2 and diisopropylethylamine (DIPEA) were distilled from CaH_2 prior to use. Organic solvents were dispensed from a solvent purification system that passes solvents through packed columns (THF and CH_2Cl_2: dry neutral alumina). HPLC-quality, acetonitrile (CH_3CN) and MilliQ water were used for HPLC analyses and purification. Chemical shifts are reported in ppm and are calibrated against residual solvent signals of $CDCl_3$ (δ 7.26, 77.2), CD_3OD (δ 3.31, 49.1) or DMSO-D_6 (δ 2.50, 39.4). All coupling constants are reported in Hz. Silica gel chromatography was performed using Merck Kieselgel Si 60. RP-HPLC analyses were performed on a Thermo system using a Chromolith performance RP-18e column (100 x 5 mm) with P1000 XR pumps. The mobile phase was a water/acetonitrile gradient with 0.1% TFA, unless otherwise noted, at a flow rate of 3 $mL{\cdot}min^{-1}$. The column effluent was monitored by UV detection at 214 and 254 nm using a Thermo UV 6000 LP diode array detector. Semi-preparative purifications of the compounds were performed on a Varian PrepStar system with SD-1 Dynamax® pumps, using a Microsorb C18 (100Å pore size, 5μ, 250*21.4 mm). The mobile phase was the same as for the analytic system, unless otherwise notified, at a flow rate of 20 $mL.min^{-1}$. The column effluent was monitored by UV detection at 214 and 254 nm using a Varian UV-Vis Prostar 325 diode array detector. Electrospray ionization (ESI) mass spectra (MS) were obtained in the positive ion mode using a LC-ToF Micromass Mass Spectrometer and matrix assisted laser desorption ionization time of flight (MALDI-Tof) mass spectra were obtained in positive ion mode using α-cyanohydroxycinnamic acid as a matrix on a Bruker Reflex II spectrometer with post source decay analysis with linear ion reflector. When MALDI was required to collect mass spectra, no exact mass measurement could be carried out. Nuclear Magnetic Resonance (NMR) spectra were recorded on a Bruker Avance 300 MHz NB US NMR spectrometer.

General procedure for methyl ester saponification. The methyl ester (typically 1.0 mmol) was dissolved in 30 mL of THF. MeOH (10 mL) was added, followed by KOH (2.5 mmol). The reaction mixture was stirred at room temperature for 12h, and then acidified with aqueous citric acid. The mixture was partitioned between CH_2Cl_2 and water. The organic layer was washed 3 times with water, dried with $MgSO_4$, filtered and evaporated to provide the product. The product was azeotroped with toluene to

remove all traces of water, characterized by ^{1}H NMR, and used in the next step without further purification.

General procedure for trimethylsilyl ethanol ester (TMSE) deprotection. The TMSE ester (typically 1 mmol) was dissolved in dry THF (10 mL). Anhydrous TBAF (5 mmol) was added dropwise at room temperature. The reaction mixture was stirred at room temperature for 12h. Reaction progress was monitored by TLC, and additional TBAF was added if necessary to complete the reaction. All volatiles were then evaporated under reduced pressure and the acid was purified by column chromatography on silica gel.

General procedure for nitro group reduction. The nitro precursor (typically 2 mmol) was dissolved in 100 mL of EtOAc, and 200 mg of 10 % wt Pd/C was carefully added under a nitrogen atmosphere. The reaction was stirred under a hydrogen atmosphere for 12 h (pressure provided by a balloon). Reaction progress was monitored by TLC and by ^{1}H NMR. Additional Pd/C and hydrogen were added if necessary to complete the reaction. Upon completion the catalyst was removed by filtration through celite and the filtrate was evaporated to provide the crude amine. The product was characterized by ^{1}H NMR and used in the next step without further purification.

General procedure for the removal of the Boc protecting groups. The Boc protected oligomer (typically 1.0 mmol) was dissolved in 4 mL of CH_2Cl_2/TFA (1:1 vol/vol) and the solution was stirred at room temperature for 2 h. The solvents were evaporated to provide the crude product. The product was purified by multiple semipreparative HPLC runs using a microsorb column (Microsorb C18, 100Å, 5µ, 250*21.4 mm) and a water/acetonitrile gradient with 0.1% TFA.

General procedure for coupling an amine and an acid. The acid (typically 1.0 mmol) was suspended in 20 mL of dry CH_2Cl_2 under an argon atmosphere and 1-chloro-*N,N*-2-trimethylpropenylamine (2.0 mmol) was added. The reaction mixture was stirred at 25 °C for 2 h resulting in a homogeneous solution, then evaporated to provide the corresponding acid chloride. To a solution of the amine (1.1 mmol) in 20 mL of dry CH_2Cl_2 containing DIPEA (3.7 mmol), the acid chloride in dry CH_2Cl_2 was added. The reaction mixture was stirred at 25°C for 12 h, then the solvent was evaporated.

Trimer 15 from dimer amine 14 and monomer acid 13. The general procedure for coupling an amine and an acid was used. The crude product was purified by silica gel chromatography using EtOAc/cyclohexane (1:1 vol/vol) to obtain compound **15** (0.25 g, 90% yield) as a yellow solid. ^{1}H NMR

(300 MHz, $CDCl_3$): δ 1.45 (bs, 27H), 2.02-2.14 (m, 2H), 2.16-2.30 (m, 4H), 3.34-3.56 (m, 6H), 3.53 (s, 3H), 4.20 (t, 2H, *J* = 5.4 Hz), 4.36 (t, 2H, *J* = 5.5 Hz), 4.44 (t, 2H, *J* = 5.9 Hz), 4.84 (bs, 3H), 4.91 (t, 2H, *J* = 5.4 Hz), 6.75 (d, 1H, *J* = 6.0 Hz), 7.46 (s, 1H), 7.65 (t, 1H, *J* = 5.0 Hz), 7.75 (t, 1H, *J* = 5.2 Hz), 7.77 (s, 1H), 7.78 (s, 1H), 7.98 (t, 2H, *J* = 4.2 Hz), 8.99 (d, 1H, *J* = 6.1 Hz), 9.04 (d, 1H, *J* = 6.3 Hz), 12.26 (s, 2H); ^{13}C NMR (75 MHz, $CDCl_3$): δ 28.3, 29.2, 37.6, 52.7, 55.2, 66.4, 79.3, 101.1, 107.1, 110.0, 115.7, 117.3, 121.8, 128.2, 134.8, 139.1, 146.9, 151.8, 155.0, 156.0, 161.0, 162.2, 165.1, 167.2; MS calcd $[M+Na]^+$ ($C_{52}H_{65}N_{11}O_{13}Na$): 1074.4680. Found: (TOF HRMS ESI) 1074.4675.

Trimer acid 15a. Ester **15** (150 mg, 0.14 mmol) was saponified to the corresponding acid **15a** in quantitative yield using the general experimental procedure with NaOH (14 mg, 0.35 mmol), THF (2 mL) and MeOH (2 mL). ^1H NMR (300 MHz, $CDCl_3$): δ 1.44 (s, 9H), 1.48 (s, 18H), 1.96-2.12 (m, 3H), 2.14-2.30 (m, 3H), 3.27-3.57 (m, 6H), 4.10 (t, 2H, *J* = 5.4 Hz), 4.20-4.47 (m, 4H), 4.40 (s, 2H), 4.67 (bs, 1H), 4.89 (bs, 2H), 6.76 (s, 1H), 6.85 (d, 1H, *J* = 6.0 Hz), 6.90 (d, 1H, *J* = 6.2 Hz), 7.17 (d, 1H, *J* = 6.0 Hz), 7.18 (s, 1H), 7.24 (d, 1H, *J* = 6.7 Hz), 7.57 (t, 1H, *J* = 4.5 Hz), 7.80 (s, 1H), 7.93 (d, 1H, *J* = 5.9 Hz), 8.93 (d, 1H, *J* = 6.0 Hz), 11.78 (s, 1H), 11.98 (s, 1H); ^{13}C NMR (75 MHz, $CDCl_3$): δ 28.3, 28.5, 29.1, 37.2, 38, 46, 49.9, 50.8, 53.2, 54, 55.2, 66, 66.8, 79.1, 99, 107.1, 108.9, 116, 117.2, 121.6, 125.1, 134.2, 137.8, 146.5, 152, 155.9, 156.0, 162, 163, 166.2, 167.2, 168.1; MS calcd $[M+Na]^+$ ($C_{51}H_{63}N_{11}O_{13}Na$): 1060.4607. Found: (TOF HRMS ESI) 1060.4604.

Cyclic Trimer 16 from trimer acid 15a. To a solution of trimer acid **15a** (70 mg, 0.067 mmol) in dry CH_2Cl_2 (2 mL) were added pentafluorophenol (24 mg, 0.13 mmol) and EDCI (25 mg, 0.13 mmol) at 0 °C. The reaction mixture was stirred for 12 h at 25 °C. Water was then added and the aqueous layer was extracted with CH_2Cl_2 (2 x 5 mL). The combined organic layers were washed with brine (5 mL), dried over anhydrous $MgSO_4$ and filtered. The filtrate was concentrated under reduced pressure. The resulting crude pentafluorophenyl ester was taken directly in THF (5 mL) and a catalytic amount of 10% Pd/C (10 mg) was carefully added under an oxygen free atmosphere. The reaction mixture was stirred under a hydrogen atmosphere at 25°C for 12 h. The reaction progress was monitored by TLC. The reaction mixture was filtered over celite. Solids were washed with CH_2Cl_2 (2 x 4 mL). Solvents were removed under reduced pressure, to obtain the crude amino-pentafluoropenyl ester which was set to react immediately without further purification. This material (70 mg, 0.05 mmol) was dissolved in dry toluene (5 mL). A catalytic amount of DMAP (5 mg) was added and the mixture was stirred at 100 °C for 4 h. Toluene was evaporated under reduced pressure. The crude product was purified by column chromatography in pure EtOAc to obtain cyclic trimer **16** (40 mg, 70% yield, in three steps) as a yellow solid. ^1H NMR (300 MHz, $CDCl_3$): δ 1.45 (s, 18H), 1.47 (m, 9H), 1.99-2.10 (m, 2H), 2.13-2.27 (m, 4H),

3.30-3.39 (m, 2H), 3.40-3.54 (m, 4H), 4.19 (t, 2H, J = 5.8 Hz), 4.33-4.46 (m, 4H), 4.78 (d, 2H, J = 6.0 Hz), 4.93 (bs, NH), 7.08 (d, 1H, J = 2.3 Hz), 7.52-7.66 (m, 2H), 7.72 (s, 1H), 7.78 (s, 1H), 7.94-8.04 (m, 3H), 8.70 (d, 1H, J = 6.2 Hz), 8.86 (d, 1H, J = 6.0 Hz), 9.09 (t, 1H, J = 7.5 Hz), 10.56 (s, 1H), 10.95 (s, 1H); ^{13}C NMR (75 MHz, $CDCl_3$): δ 28.3, 28.4, 29.5, 31.4, 36.5, 37.4, 37.7, 43.9, 61.7, 66.2, 66.6, 77.2, 79.3, 98.4, 100.1, 107.7, 111.7, 116.6, 117.2, 119.8, 121.2, 121.4, 122.4, 126.3, 126.7, 133.3, 133.9, 138.7, 149.2, 150.5, 152.7, 156.1, 156.2, 157.7, 161.4, 161.9, 162.6, 162.8, 163.2, 163.9, 166.9; HRMS calcd $[M+H]^+$ ($C_{51}H_{64}N_9O_{12}$): 994.4669. Found (TOF HRMS ESI) 994.4665.

Compound 6 from cyclic trimer 16. Compound **6** was prepared from cyclic trimer **16** (20 mg, 0.02 mmol) using the general procedure for the removal of Boc protecting groups. The crude product showed a single peak in analytical HPLC (chromolith C18 column, solvents: H_2O containing 0.1% TFA). Quantitative yield of a light yellow solid. ^{1}H NMR (300 MHz, CD_3OD): δ 2.02-2.10 (m, 2H), 2.22-2.48 (m, 4H), 3.04-3.17 (m, 2H), 3.17-3.27 (m, 4H), 4.04-4.27 (m, 4H), 4.44-4.58 (m, 2H), 4.73-4.87 (m, 2H), 6.37 (bs, 1H), 6.91-7.25 (m, 4H), 7.29-7.58 (m, 3H), 7.87 (s, 1H), 7.98 (s, 1H), 8.32 (d, 1H, J = 1.70 Hz), 9.13 (bs, 1H), 9.65 (bs, 1H); MS calcd $[M+H)^+$ ($C_{36}H_{40}N_9O_6$): 694.3023; Found: (TOF HRMS ESI) 694.3020.

Tetramer methyl amide 18 from tetramer ester 17. A solution of tetramer **17** (70 mg, 0.048 mmol), methylamine (excess amount, 100 equiv.) in a mixture of THF/MeOH (2 mL, 5:1 vol/vol) was stirred at room temperature for 24 h. The solvents were evaporated, then water was added. The aqueous layer was extracted with CH_2Cl_2 (2 x 5 mL). The combined organic layers were dried over $MgSO_4$, filtered, and evaporated under reduced pressure. The crude product was purified by column chromatography, EtOAc/cyclohexane (4:1) to afford **18** (56 mg, 80% yield) as a yellow solid. ^{1}H NMR (300 MHz, $CDCl_3$): δ 1.42 (s, 18H), 1.49 (s, 18H), 2.14-2.29 (m, 6H), 2.32-2.43 (m, 2H), 2.63 (d, 3H, J = 8.3 Hz), 3.37-3.72 (m, 8H), 4.13-4.24 (m, 6H), 4.44-4.66 (m, 2H), 4.70 (bs, 1H), 4.90 (bs, 1H), 5.51 (bs, 1H), 6.39 (bs, 1H), 6.75 (s, 1H), 6.83 (s, 1H), 7.27 (t, 1H, J = 8.1 Hz), 7.39 (t, 1H, J = 8.3 Hz), 7.52 (s, 1H), 7.54 (t, 1H, J = 7.4 Hz), 7.60 (t, 1H, J = 7.74 Hz), 7.76 (t, 1H, J = 7.7 Hz), 7.80-8.05 (m, 6H), 8.50 (d, 2H, J = 7.9 Hz), 9.03 (d, 1H, J = 7.3 Hz), 11.60 (s, 1H), 11.63 (s, 1H), 12.26 (s, 1H); ^{13}C NMR (75 MHz, $CDCl_3$): δ 28.6, 29.1, 29.2, 29.3, 29.6, 37.3, 37.5, 38.0, 38.3, 52.5, 66.5, 66.8, 67.3, 79.3, 79.5, 79.6, 79.8, 97.7, 98.8, 100.2, 100.7, 116.0, 116.3, 116.7, 116.8, 117.1, 118.0, 121.9, 121.2, 120.0, 123.7, 124.7, 125.9, 127.1, 127.7, 127.9, 128.6, 133.8, 134.1, 135.4, 138.4, 139.1, 139.2, 145.5, 148.8, 151.1, 153.9, 156.9, 156.4, 156.5, 161.8, 161.4, 162.2, 162.9, 163.1, 163.6, 164.5; MS calcd [M+H]+ ($C_{73}H_{87}N_{13}O_{18}Na$): 1456.1034 Found: (TOF HRMS ESI) 1456.1030.

Compound 8 from tetramer amide 18. Compound **8** was produced from tetramer amide **18** (20 mg, 0.013 mmol) using the general procedure for the removal of Boc protecting groups. The crude product was purified by reverse phase HPLC, eluting with a pure water to pure CH_3CN gradient containing 0.1% TFA, to obtain **8** (quantitative yield) as a yellow solid. ^1H NMR (300 MHz, CD_3OD): δ 2.30-2.45 (m, 4H), 2.46 (d, 3H, *J* = 8.3 Hz), 2.46-2.59 (m, 4H), 3.26-3.41 (m, 4H), 3.41-3.51 (m, 4H), 4.03-4.14 (m, 2H), 4.15-4.25 (m, 2H), 4.58-4.71 (m, 4H), 6.37 (s, 1H), 6.45 (s, 1H), 7.18 (t, 1H, *J* = 8.1 Hz), 7.32 (t, 1H, *J* = 8.3 Hz), 7.34 (s, 1H), 7.42 (t, 1H, *J* = 8.3 Hz), 7.50 (t, 1H, *J* = 8.3 Hz), 7.63-7.73 (m, 3H), 7.78 (s, 1H), 7.79 (d, 2H, *J* = 7.9 Hz), 7.99 (d, 1H, *J* = 7.9 Hz), 8.24 (d, 1H, *J* = 7.9 Hz), 8.50 (d, 1H, *J* = 7.9 Hz), 8.83 (d, 1H, *J* = 7.3 Hz), 11.28 (s, 1H), 11.39 (s, 1H), 12.01 (s, 1H); MS calcd [M+H]+ ($C_{53}H_{56}N_{13}O_{10}$): 1034.4195; Found: (TOF HRMS ESI) 1034.4191.

Compound 7 from ester 17: Compound was **7** obtained from tetramer ester **17** (50 mg, 0.034 mmol) using the general procedure for the removal of Boc protecting groups. The crude product was purified by reverse phase HPLC eluting with a pure water to pure CH_3CN gradient containing 0.1% TFA to provide **7** (quantitative yield) as a yellow solid. ^1H NMR (300 MHz, CD_3OD): δ 2.32-2.65 (m, 8H), 3.27-3.51 (m, 8H), 3.52 (s, 3H), 4.24-4.41 (m, 4H), 4.62-4.73 (m, 4H), 6.78 (s, 1H), 6.93 (s, 1H), 7.45 (t, 1H, *J* = 8.3 Hz), 7.54 (s, 1H), 7.58-7.68 (m, 2H), 7.73-7.85 (m, 2H), 7.94 (s, 1H), 7.97-8.11 (m, 4H), 8.48 (d, 1H, *J* = 8.0 Hz), 8.64 (d, 1H, *J* = 8.0 Hz), 9.09 (d, 1H, *J* = 1.3 Hz), 11.79 (s, 1H), 11.97 (s, 1H), 12.30 (s, 1H); MS calcd [M+H]+ ($C_{53}H_{55}N_{12}O_{11}$): 1035.4056; Found: (TOF HRMS ESI) 1035.4050.

Compound 9 from tetramer 7. To a stirred, cooled solution of tetramer **7** (20 mg, 0.014 mmol), 1-H-pyrazol-1-carboxamidine hydrochloride (30 mg, 0.021 mmol) in dry DMF (2 mL) was added, followed by Et_3N (30 mg, 0.23 mmol). The reaction mixture was stirred at 25°C for 12h. The DMF was evaporated and the crude product was directly treated with TFA/DCM (1:1) for 4 h. After evaporation of solvents, the product was purified by reverse phase HPLC eluting with a pure water to pure CH_3CN gradient containing 0.1% TFA, to provide **9** (10 mg, 60% yield) as a yellow solid. ^1H NMR (300 MHz, CD_3OD): d 2.30-2.61 (m, 8H), 3.52 (s, 3H), 3.60-3.70 (m, 6H), 3.71-3.80 (m, 2H), 4.24-4.40 (m, 4H), 4.58-4.75 (m, 4H), 6.79 (s, 1H), 6.95 (s, 1H), 7.45 (t, 1H, *J* = 7.9 Hz), 7.53 (s, 1H), 7.59-7.68 (m, 2H), 7.73-7.85 (m, 2H), 7.95 (s, 1H), 8.01-8.15 (m, 4H), 8.48 (d, 1H, *J* = 8.3 Hz), 8.66 (d, 1H, *J* = 7.9 Hz), 9.09 (d, 1H, *J* = 7.7 Hz), 11.72 (s, 1H), 11.97 (s, 1H), 12.30 (s, 1H); MS calcd $[M+H]^+$ ($C_{57}H_{62}N_{20}O_{11}$): 1203.4907; Found: (MALDI MS) 1203.4902.

Compound 10 from tetramer 19: Compound **10** was derived from **19** (20 mg, 0.014 mmol) using the general procedure for the removal of the Boc protecting groups. The crude product was purified by

reverse phase HPLC eluting with a pure water to pure CH_3CN gradient containing 0.1% TFA, to obtain **10** (12 mg, 95% yield). ^{1}H NMR (300 MHz, CD_3OD): δ 2.25-2.47 (m, 8H), 3.22-3.40 (m, 8H), 3.45 (s, 3H), 4.27 (t, 2H, *J* = 5.28 Hz), 4.36-4.52 (m, 8H), 5.06 (s, 2H), 6.77 (s, 1H), 7.20 (d, 1H, *J* = 1.7 Hz), 7.26 (d, 1H, *J* = 1.3 Hz), 7.40 (s, 1H), 7.42 (t, 1H, *J* = 8.3 Hz), 7.54 (t, 1H, *J* = 8.1 Hz), 7.67 (d, 1H, *J* = 1.7 Hz), 7.68 (s, 1H), 7.83 (d, 1H, *J* = 8.3 Hz), 7.90 (d, 1H, *J* = 8.3 Hz), 8.24 (t, 2H, *J* = 7.4 Hz), 9.69 (t, 1H, *J* = 3.6 Hz), 11.34 (s, 2H); MS calcd [M+H]+ ($C_{47}H_{56}N_{12}O_9$): 932.43; Found: (TOF MS ESI) 932.40.

Dimer 22a from acid 20 and amine 21. Dimer **22a** was obtained from acid **20** (0.95 g, 2.44 mmol) and amine **21** (1.02 g, 2.44 mmol) using the general procedure for coupling an amine and an acid. The crude product was recrystallized from MeOH to yield dimer **22a** (1.60 g, 85% yield) as a yellow solid. ^{1}H NMR (300 MHz, $CDCl_3$): δ 0.07 (s, 9H), 1.27 (t, 2H, *J* = 8.3 Hz), 1.44 (s, 9H), 1.52 (s, 9H), 2.19-2.29 (m, 2H), 3.43-3.53 (m, 2H), 4.46 (t, 2H, *J* = 5.8 Hz), 4.73 (t, 2H, *J* = 8.3 Hz), 4.84 (s, 2H), 7.50 (s, 1H), 7.66 (t, 1H, *J* = 8.5 Hz), 7.67 (t, 1H, *J* = 7.3 Hz), 7.95 (s, 1H), 8.09 (d, 1H, *J* = 8.4 Hz), 8.20 (d, 1H, *J* = 7.7 Hz), 8.52 (d, 1H, *J* = 8.2 Hz), 9.06 (d, 1H, *J* = 7.0 Hz), 11.87 (s, 1H); ^{13}C NMR (75 MHz, $CDCl_3$): δ 0.00, 19.11, 29.52, 30.04, 66.34, 67.24, 68.67, 77.30, 84.72, 101.72, 102.98, 118.32, 120.42, 123.41, 124.75, 127.06, 128.19, 129.61, 136.37, 140.87, 141.42, 149.31, 150.24, 155.50, 157.45, 163.08, 163.94, 164.34, 167.40, 167; MS calcd $[M+H]^+$ ($C_{39}H_{50}N_5O_{11}Si$): 792.3276; Found (TOF HRMS ESI) 792.3270.

Dimer amine 22b from nitro precursor 22a. Dimer amine **22b** was obtained from dimer **22a** (0.50 g, 0.63 mmol) using the general procedure for nitro group reduction, in quantitative yield as a yellow solid. ^{1}H NMR ($CDCl_3$, 300 MHz): δ 0.00 (s, 9H), 1.10 (t, 2H, *J* = 8.1 Hz), 1.46 (s, 9H), 1.50 (s, 9H), 2.20-2.32 (m, 2H), 3.42-3.50 (m, 2H), 4.30-4.42 (m, 2H), 4.45 (t, 2H, *J* = 7.5 Hz), 4.96 (s, 2H), 5.00 (bs, 1H), 5.56 (br s, 2H), 7.00 (d, 1H, *J* = 7.6 Hz), 7.38 (t, 1H, *J* = 7.8 Hz), 7.50 (d, 1H, *J* = 8.3 Hz), 7.56 (s, 1H), 7.66 (t, 1H, *J* = 8.1 Hz), 7.76 (s, 1H), 7.90 (d, 1H, *J* = 8.3 Hz), 9.04 (d, 1H, *J* = 7.6 Hz), 12.67 (s, 1H).

Dimer acid 22c from ester 22a. Dimer ester **22a** afforded dimer acid **22c** (1.0 g, 1.26 mmol) using the general procedure for the trimethylsilylethyl ester deprotection. The crude product was purified by column chromatography eluting with CH_2Cl_2/MeOH (98:2 vol/vol) to provide **22c** (0.83 g, 95% yield) as a light yellow solid. ^{1}H NMR (300 MHz, $CDCl_3$): δ 1.45 (s, 9H), 1.53 (s, 9H), 2.20-2.30 (m, 2H), 3.44-3.53 (m, 2H), 4.48 (t, 2H, *J* = 5.8 Hz), 4.90 (s, 2H), 7.70 (s, 1H), 7.70 (t, 1H, *J* = 7.3 Hz), 7.73 (t, 1H, *J* = 7.0 Hz), 7.96 (s, 1H), 8.15 (d, 1H, *J* = 8.4 Hz), 8.27 (d, 1H, *J* = 7.7 Hz), 8.54 (, 1H, *J* = 8.4 Hz), 9.17 (d, 1H, *J* = 7.0 Hz), 11.72 (s, 1H); ^{13}C NMR (75 MHz, $CDCl_3$): δ 19.11, 28.47,58.89, 65.65, 67.33, 77.32, 83.17, 100.08, 100.87, 117.00, 118.88, 121.84, 123.10, 125.40, 125.71, 126.94, 127.60, 134.32, 139.05,

139.17, 147.24, 153.90, 155.98, 161.72, 162.38, 166.48; MS calcd ($C_{34}H_{38}N_5O_{11}$): 692.2568; Found (TOF HRMS ESI) 692.2564.

Tetramer 23a from acid 22c and amine 22b. Tetramer **23a** was obtained from dimer acid **22c** (0.39 g, 0.56 mmol) and dimer amine **22b** (0.43 g, 0.56 mmol) using the general procedure for coupling an amine and an acid. The crude product was purified by silica gel chromatography eluting with EtOAc/cyclohexane (1:1) to give **23a** (0.68 g, 85% yield) as a yellow solid. ^{1}H NMR (300 MHz, $CDCl_3$): δ -0.22 (s, 9H), 0.99 (t, 2H, *J* = 8.3 Hz), 1.47 (s, 9H), 1.54 (s, 18H), 1.60 (s, 9H), 2.11-2.24 (m, 4H), 3.31-3.42 (m, 4H), 4.11-4.22 (m, 4H), 4.30-4.45 (m, 2H), 4.91 (s, 4H), 6.56 (s, 1H), 6.81 (s, 1H), 7.33 (t, 1H, *J* = 7.3 Hz), 7.41 (t, 1H, *J* = 7.3 Hz), 7.49 (s, 1H), 7.52 (t, 1H, *J* = 7.7 Hz), 7.59-7.76 (m, 3H), 7.91 (d, 1H, *J* = 8.4 Hz), 7.99 (d, 1H, *J* = 8.4 Hz), 8.07 (d, 2H, *J* = 8.4 Hz), 8.50 (d, 2H, *J* = 8.4 Hz), 9.08 (d, 1H, *J* = 7.7 Hz), 11.64 (s, 1H), 11.91 (s, 1H), 12.28 (s, 1H); ^{13}C NMR (75 MHz, $CDCl_3$): δ 0.02, 17.12, 27.9, 29.52, 30.04, 66.34, 67.24, 68.67, 77.30, 84.72, 101.72, 102.98, 118.32, 120.42, 123.41, 124.75, 127.06, 128.19, 129.61, 136.37, 140.87, 141.42, 149.31, 150.24, 155.50, 157.45, 163.08, 163.94, 164.34, 167.40, 167; MS calcd $(M+H)^+$ ($C_{73}H_{87}N_{10}O_{19}Si$): 1435.5925; found (HRMS ESI) 1435.5918.

Tetramer amine 23b from nitro precursor 23a. Tetramer amine **23b** was obtained from tetramer **23a** (0.40 g, 0.27 mmol) using the general procedure for nitro group reduction in quantitative yield as a yellow solid. The product was considered as an intermediate and was used immediately without further characterization. ^{1}H NMR ($CDCl_3$, 300 MHz): δ -0.20 (s, 9H), 0.89 (t, 2H, *J* = 7.8 Hz), 1.39-1.53 (m, 36H), 2.10-2.32 (m, 4H), 3.38-3.68 (m, 4H), 3.79-3.86 (m, 2H), 4.04-4.18 (m, 2H), 4.38-4.48 (m, 2H), 4.50-4.64 (bs, 1H), 4.99 (bs, 4H), 5.39-5.46 (m, 1H), 5.74 (bs, 2H), 6.58 (s, 1H), 6.72 (s, 1H), 6.93 (t, 1H, *J* = 7.7 Hz), 7.11-7.24 (m, 3H), 7.36 (d, 1H, *J* = 7.8 Hz), 7.57-7.70 (m, 4H), 7.85-7.89 (m, 3H), 8.42 (d, 1H, *J* = 7.3 Hz), 8.87 (d, 1H, *J* = 7.3 Hz), 11.66 (s, 1H), 11.79 (s, 1H), 12.26 (s, 1H).

Hexamer 24a from tetramer amine 23b and dimer acid 22c. Hexamer **24a** was obtained from dimer acid **22c** (0.19 g, 0.27 mmol) and tetramer amine **23b** (0.39 g, 0.27 mmol) using the general procedure for coupling an amine and an acid. The crude product was purified by silica gel chromatography eluting with EtOAc/cyclohexane (6:4 vol/vol) to obtain hexamer **24a** (0.460 g, 80% yield) as a yellow solid. ^{1}H NMR (300 MHz, $CDCl_3$): δ -0.39 (s, 9H), 0.44 (t, 2H, *J* = 8.3 Hz), 1.47 (s, 18H), 1.54 (s, 18H), 1.57 (s, 9H), 1.60 (s, 9H), 2.15-2.41 (m, 6H), 3.40-3.62 (m, 6H), 3.74 (t, 2H, *J* = 8.3 Hz), 4.18-4.58 (m, 6H), 4.67 (s, 4H), 4.87-5.00 (m, 3 H), 5.09 (s, 2H), 5.80 (s, 1H), 6.41 (s, 1H), 6.54 (s, 1H), 6.69 (s, 2H), 6.72 (s, 1H), 7.19 (s, 1H), 7.13-7.48 (m, 6H), 7.68 (t, 1H, *J* = 7.7 Hz), 7.72 (d, 1H, *J* = 8.0 Hz), 7.80 (d, 1H, *J* = 7.2 Hz), 7.84-7.92 (m, 3H), 7.96-8.04 (m, 2H), 8.12-8.18 (m, 1H), 8.25 (d, 1H, *J* = 7.3 Hz), 8.30 (d, 1H, *J* = 8.1 Hz), 8.50-8.58 (m, 1H), 11.33 (s, 1H), 11.38 (s, 1H), 11.64 (s, 2H), 11.81 (s, 1H); ^{13}C NMR (75

MHz, $CDCl_3$): δ -0.24, 17.04, 29.52, 29.60, 29.85, 30.20, 30.45, 38.04, 38.74, 64.30, 66.18, 66.89, 66.98, 67.24, 68.67, 78.26, 78.90, 80.15, 80.24, 83.82, 83.93, 83.99, 98.50, 98.64, 98.94, 100.56, 100.97, 115.5, 116.1, 116.3, 116.7, 116.9, 117.0, 121.4, 121.7, 121.9, 122.3, 123.4, 124.3, 126.0, 126.3, 126.6, 127.2, 127.5, 127.9, 133.3, 134.2, 134.3, 134.4, 134.5, 134.8, 138.4, 138.8, 138.9, 139.1, 139.5, 139.8, 144.9, 145.0, 148.5, 148.8, 149.0, 150.6, 153.1, 156.2, 156.3, 156.5, 159.9, 160.3, 160.6, 161.0, 161.2, 161.8, 162.6, 162.8, 162.9, 163.0, 163.3, 164.0, 167.0, 167.2, 167.5; MS calcd [M+H]+ ($C_{107}H_{124}N_{15}O_{27}Si$): 2078.85. Found: (MALDI MS) 2078.81.

Hexamer amine 24b from nitro precursor 24a. Hexamer amine **24b** was obtained from hexamer **24a** (0.30 g, 0.14 mmol) using the general procedure for nitro group reduction (0.28 g, 95% yield) as a yellow solid. The product was considered as an intermediate and was used immediately without further characterization. ^1H NMR ($CDCl_3$, 300 MHz): δ -0.21 (s, 9H), 0.85 (t, 2H, *J* = 8.0 Hz), 1.36-1.52 (m, 54H), 2.00-2.32 (m, 6H), 3.16-3.46 (m, 6H), 4.00-4.39 (m, 6H), 4.52-4.59 (m, 2H), 4.64-4.72 (m, 2H), 4.74 (s, 2H), 4.80 (s, 4H), 5.82 (d, 1H, *J* = 7.2 Hz), 6.34 (s, 1H), 6.46 (s, 1H), 6.71 (s, 1H), 6.75 (s, 1H), 6.85 (s, 1H), 6.92-7.02 (m, 2H), 7.08-7.16 (m, 3H), 7.28-7.46 (m, 3H), 7.56 (t, 1H, *J* = 8.1 Hz), 7.70 (d, 1H, *J* = 8.3 Hz), 7.74 (d, 1H, *J* = 7.6 Hz), 7.82-7.88 (m, 4H), 7.92-7.97 (m, 2H), 8.15 (d, 1H, *J* = 7.6 Hz), 8.58 (d, 1H, *J* = 7.3 Hz), 11.22 (s, 1H), 11.26 (s, 1H), 11.42 (s, 2H), 11.64 (s, 1H).

Octamer 25a from dimer acid 22c and hexamer amine 24b. Octamer **25a** was obtained from dimer acid **22c** (0.10, 0.14 mmol) and hexamer amine **24b** (0.28 g, 0.14 mmol) using the general procedure for coupling an amine and an acid. The crude product was purified by silica gel chromatography eluting with EtOAc/cyclohexane (6:4 vol/vol) to obtain **25a** (0.30 g, 80% yield) as a yellow solid. ^1H NMR (300 MHz, DMSO-d_6): δ -0.51 (s, 9H), 0.28 (t, 2H, *J* = 8.3 Hz), 1.40 (s, 18H), 1.45 (s, 9H), 1.48 (s, 9H), 1.50 (s, 9H), 1.53 (s, 9H), 1.63 (s, 9H), 1.75 (s, 9H), 2.03-2.18 (m, 6H), 2.27 (m, 2H), 3.19-3.37 (m, 6H), 3.4-3.65 (m, 4H), 4.07-4.26 (m, 4H), 4.28-4.42 (m, 4H), 4.61 (d, 1H, *J* = 6.3 Hz), 4.73-5.05 (m, 6H), 5.20 (d, 1H, *J* = 6.5 Hz), 5.99 (s, 1H), 6.21 (s, 1H), 6.24 (s, 1H), 6.27 (s, 1H), 6.49 (s, 1H), 6.57 (s, 1H), 6.84 (s, 1H), 6.91 (s, 1H), 7.01-7.26 (m, 5H), 7.31-7.41 (m, 7H), 7.50-7.72 (m, 3H), 7.75-7.95 (m, 5H), 8.06 (d, 1H, *J* = 8.4 Hz), 8.20 (d, 1H, *J* = 8.0 Hz), 8.30 (d, 1H, *J* = 8.4 Hz), 8.32 (d, 1H, *J* = 8.2 Hz), 10.73 (s, 1H), 10.83 (s, 1H), 10.87 (s, 2H), 10.99 (s, 1H), 11.09 (s, 1H), 11.29 (s, 1H); ^{13}C NMR (75 MHz, $CDCl_3$): δ -0.60, 17.57, 27.12, 29.61, 29.76, 29.84, 30.04, 30.25, 30.33, 30.36, 30.38, 30.95, 30.96, 39.10, 64.64, 67.36, 67.93, 68.02, 68.54, 68.68, 69.02, 69.11, 79.59, 79.73, 84.06, 84.12, 84.20, 84.27, 99.45, 99.56, 99.68, 100.06, 100.53, 101.47, 101.05, 102.18, 116.83, 116.95, 117.09, 117.23, 117.58, 118.07, 118.14, 118.61, 122.44, 122.49, 122.86, 122.22, 123.31, 123.47, 123.53, 124.85, 127.15, 127.89, 128.12, 128.54, 128.93, 129.89, 129.98, 133.91, 133.97, 134.01, 134.73, 135.13, 135.21, 138.60, 138.71, 138.75, 139.15, 139.20,

129.64, 140.00, 146.22, 146.79, 149.80, 149.86, 150.05, 150.188, 151.48, 154.17, 157.73, 157.83, 157.87, 160.15, 160.22, 160.74, 161.71, 161.89, 162.35, 163.23, 163.32, 163.72, 163.87, 163.96, 164.00, 164.20, 164.34, 168.42, 168.45, 168.65, 168.70; MS calcd $[M+H]^+$ ($C_{141}H_{161}N_{20}O_{35}Si$): 2722.11. Found: (MALDI MS) 2722.10.

Octamer acid 25b from ester 25a. Octamer ester **25a** afforded octamer acid **25b** (1.0 g, 0.03 mmol) using the general procedure for the trimethylsilylethyl ester deprotection. The crude product was purified by column chromatography eluting with CH_2Cl_2/MeOH/AcOH (97:2:1 vol/vol/vol) to provide **25b** (81 mg, 85% yield) as a yellow solid. ^1H NMR (300 MHz, DMSO-d_6): δ 1.23 (s, 9H), 1.37-1.55 (m, 45H), 1.68 (s, 9H), 1.76 (s, 9H), 2.05-2.19 (m, 6H), 2.25-2.34 (m, 2H), 3.19-3.38 (m, 6H), 3.43-3.50 (m, 2H), 3.93-4.02 (m, 2H), 4.09-4.27 (m, 4H), 4.30-4.44 (m, 2H), 4.57-5.04 (m, 8H), 5.17 (bs, 1H), 5.23 (bs, 1H), 6.03 (s, 1H), 6.27 (s, 1H), 6.28 (s, 1H), 6.29 (s, 1H), 6.30 (s, 2H), 6.52 (s, 1H), 6.58 (s, 1H), 6.85 ((d, 1H, *J* = 8.4 Hz), 6.91-7.00 (m, 2H), 7.08-7.27 (m, 3H), 7.31-7.47 (m, 7H), 7.52 (t, 1H, *J* = 8.4 Hz), 7.61-7.73 (m, 2H), 7.73-7.96 (m, 5H), 8.06 (d, 1H, *J* = 8.4 Hz), 8.12 (d, 1H, *J* = 8.0 Hz), 8.30 (d, 1H, *J* = 7.l6 Hz), 10.78 (s, 1H), 10.82 (s, 1H), 10.88 (s, 2H), 10.02 (s, 1H), 11.13 (s, 1H), 11.21 (s, 1H); ^{13}C NMR (75 MHz, $CDCl_3$): δ 17.6, 27.15, 29.62, 29.70, 29.80, 30.0, 30.22, 30.35, 30.36, 30.38, 30.98, 30.96, 39.10, 64.64, 67.36, 67.93, 68.02, 68.54, 68.68, 69.02, 69.11, 79.59, 79.73, 84.06, 84.12, 84.20, 84.27, 99.45, 99.56, 99.68, 100.06, 100.53, 101.47, 101.05, 102.18, 116.83, 116.95, 117.09, 117.23, 117.58, 118.07, 118.14, 118.61, 122.44, 122.49, 122.86, 122.22, 123.31, 123.47, 123.53, 124.85, 127.15, 127.89, 128.12, 128.54, 128.93, 129.89, 129.98, 133.91, 133.97, 134.01, 134.73, 135.13, 135.21, 138.60, 138.71, 138.75, 139.15, 139.20, 129.64, 140.00, 146.22, 146.79, 149.80, 149.86, 150.05, 150.188, 151.48, 154.17, 157.73, 157.83, 157.87, 160.15, 160.22, 160.74, 161.71, 161.89, 162.35, 163.20, 163.32, 163.52, 163.83, 163.92, 164.04, 164.25, 164.34, 168.40, 168.42, 168.6; MS calcd $[M+H]^+$ ($C_{136}H_{149}N_{20}O_{35}$): 2622.04. Found: (MALDI MS) 2622.00.

Compound 11 from octamer acid 25b. Compound **11** was obtained from octamer acid **25b** (20 mg, 0.014 mmol) using the general procedure for the removal of Boc protecting groups. The crude product was purified by multiple semipreparative HPLC runs using a C18 column eluting with a pure water to pure CH_3CN gradient containing 0.1% TFA to give **11** (quantitative yield) as a yellow solid. ^1H NMR (300 MHz, CD_3OD/DMSO-D_6 1:1 vol/vol): δ 2.21-2.38 (m, 8H), 3.33-3.44 (m, 8 H), 3.97-4.17 (m, 2H), 4.19-4.31 (m, 2H), 4.36-4.47 (m, 4H), 4.59-4.79 (m, 4H), 4.89-5.16 (m, 4H), 6.01 (s, 1H), 6.18 (s, 1H), 6.23 (s, 1H), 6.29 (s, 1H), 6.49 (s, 1H), 6.52 (s, 1H), 6.85 (s, 1H), 6.90 (s, 1H), 7.09 (d, 1H, *J* = 7.73 Hz), 7.18 (t, 1H, *J* = 7.74 Hz), 7.30-7.59 (m, 10H), 7.61-7.98 (m, 9H), 8.05 (d, 1H, *J* = 7.98 Hz), 8.13 (d, 1H, *J*

= 7.98 Hz), 8.33 (d, 1H, *J* = 6.61 Hz), 10.79 (s, 1H), 10.81 (s, 1H), 10.84 (s, 1H), 10.85 (s, 1H), 10.95 (s, 1H), 11.16 (s, 2H); MS calcd $[M+H]^+$ ($C_{100}H_{85}N_{20}O_{27}$): 1997.58. Found: (MALDI MS) 1997.54.

Compound 12 from octamer acid 26. Octamer acid **26** (30 mg, 0.0114 mmol) was treated using the general procedure for the removal of Boc protecting groups. The crude product was purified by multiple semipreparative HPLC runs using a C18 column eluting with a pure water to pure CH_3CN gradient containing 0.1% TFA to give **12** (22 mg, 80% yield) as a yellow solid. ^{1}H NMR (300 MHz, DMSO-d_6): δ2.25-2.40 (m, 8H), 3.18 -3.65 (m, 8H), 4.18-4.59 (m, 8H), 4.60-5.00 (m, 8H), 5.96 (s, 1H), 6.29 (s, 1H), 6.40 (s, 1H), 6.50 (s, 1H), 6.92 (s, 1H), 7.02 (s, 1H), 7.05-7.70 (m, 21H), 7.76-7.95 (m, 6H), 8.07 (m, 2H), 8.32 (d, 1H, *J* = 7.2 Hz), 10.76 (s, 1H), 10.89 (m, 3H), 11.02 (s, 1H), 11.06 (s, 1H), 11.13 (s, 1H); MS calcd $[M+H]^+$ ($C_{100}H_{85}N_{20}O_{27}$): 1997.58; Found: (MS MALDI) 1997.50.

3. References

1 E. R. Gillies, F. Deiss, C. Staedel, J.-M. Schmitter, I. Huc, *Angew. Chem. Int. Ed.* **2007**, *46*, 4081–4084.
2 S. P. Shirude, E. R. Gillies, S. Ladame, F. Godde, K. Shin-ya, I. Huc, S. Balasubramanian, *J. Am. Chem. Soc.* **2007**, *129*, 11890–11891.
3 B. Baptiste, Céline Douat-Casassus, K. Laxmi-Reddy, F Godde, I. Huc, *J. Org. Chem.* **2010**, *75*, 7175–7185.
4 L. Delaurière, Z. Dong, K. Laxmi-Reddy, F. Godde, J.-J. Toulmé, I. Huc, *Angew. Chem. Int. Ed.* **2012**, *51*, 473–477.

4. ^{1}H NMR spectra and chromatograms

Compound 15 (300 MHz, $CDCl_3$)

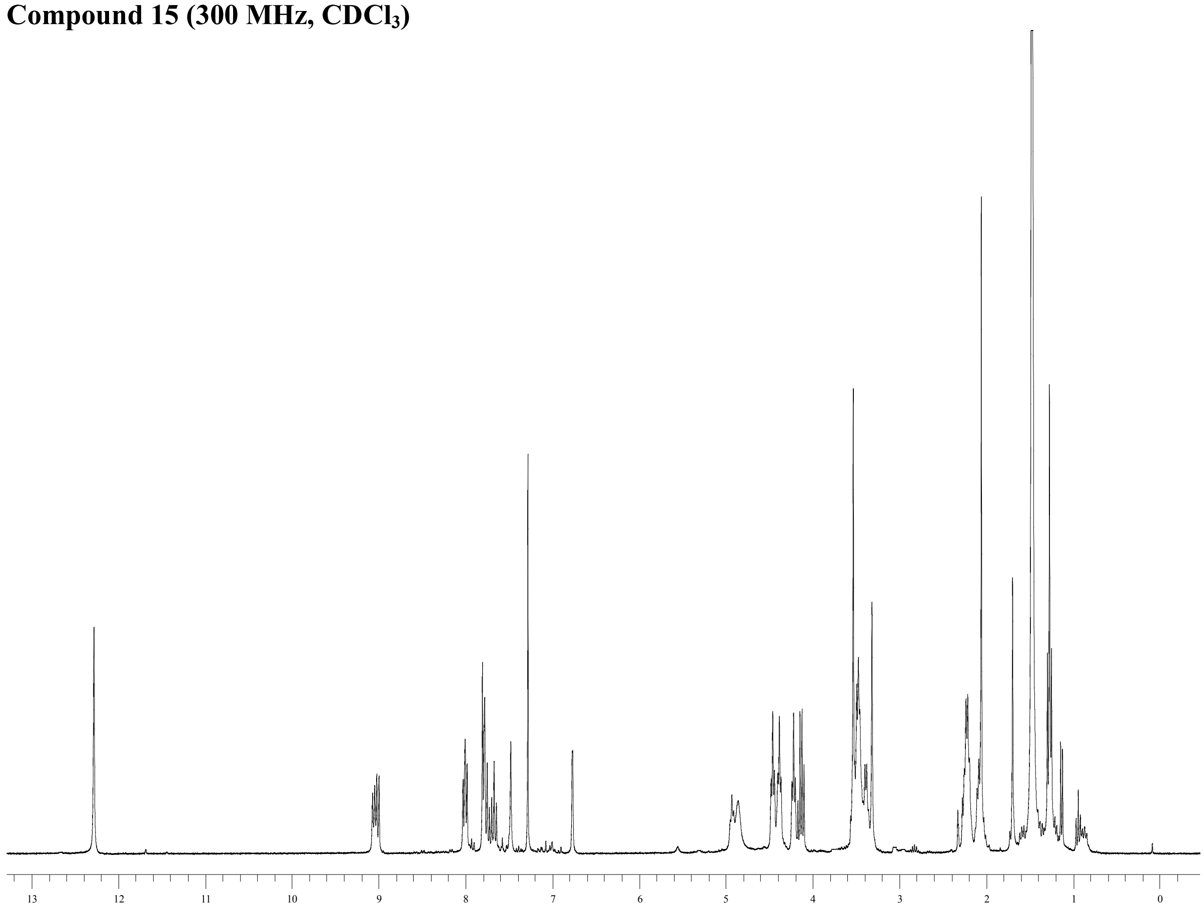

Compound 16 (300 MHz, $CDCl_3$)

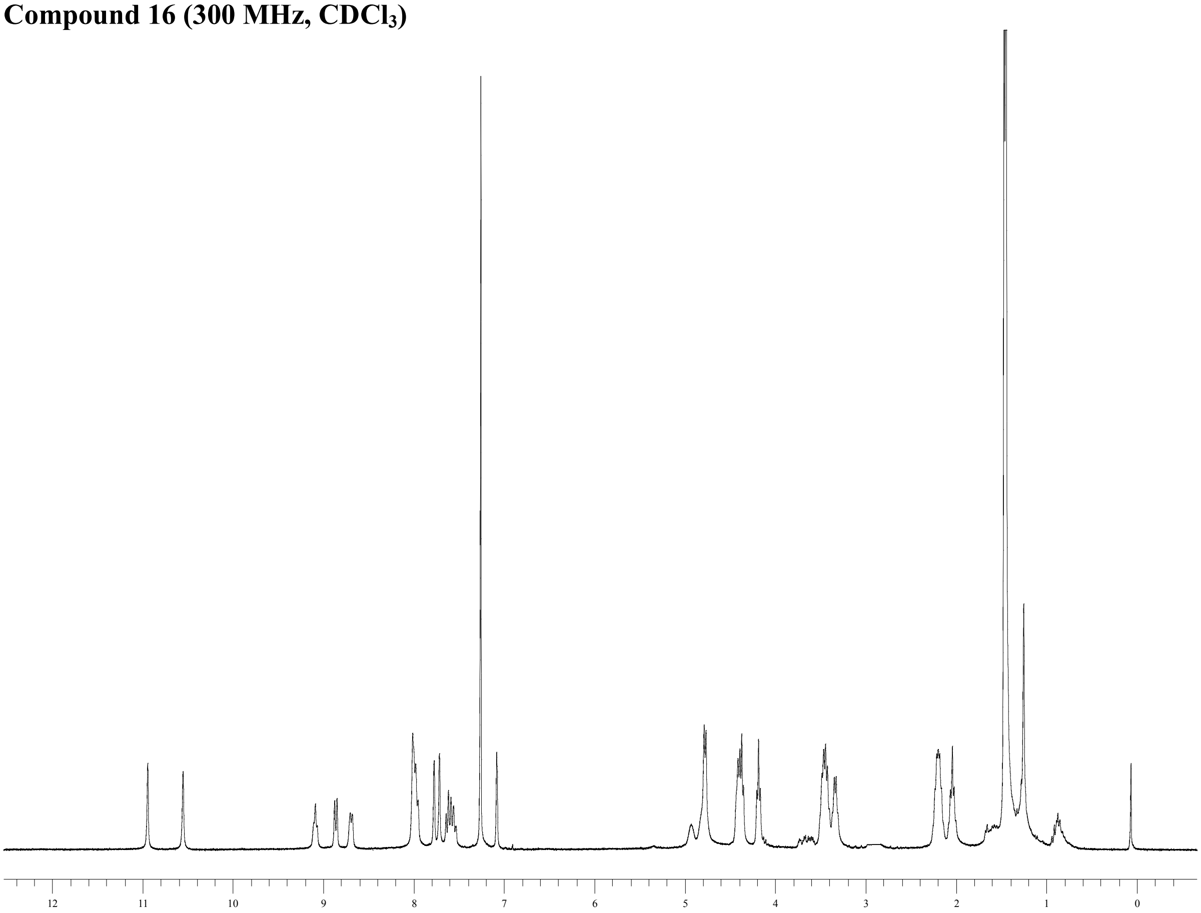

Compound 6 (300 MHz, CD_3OD)

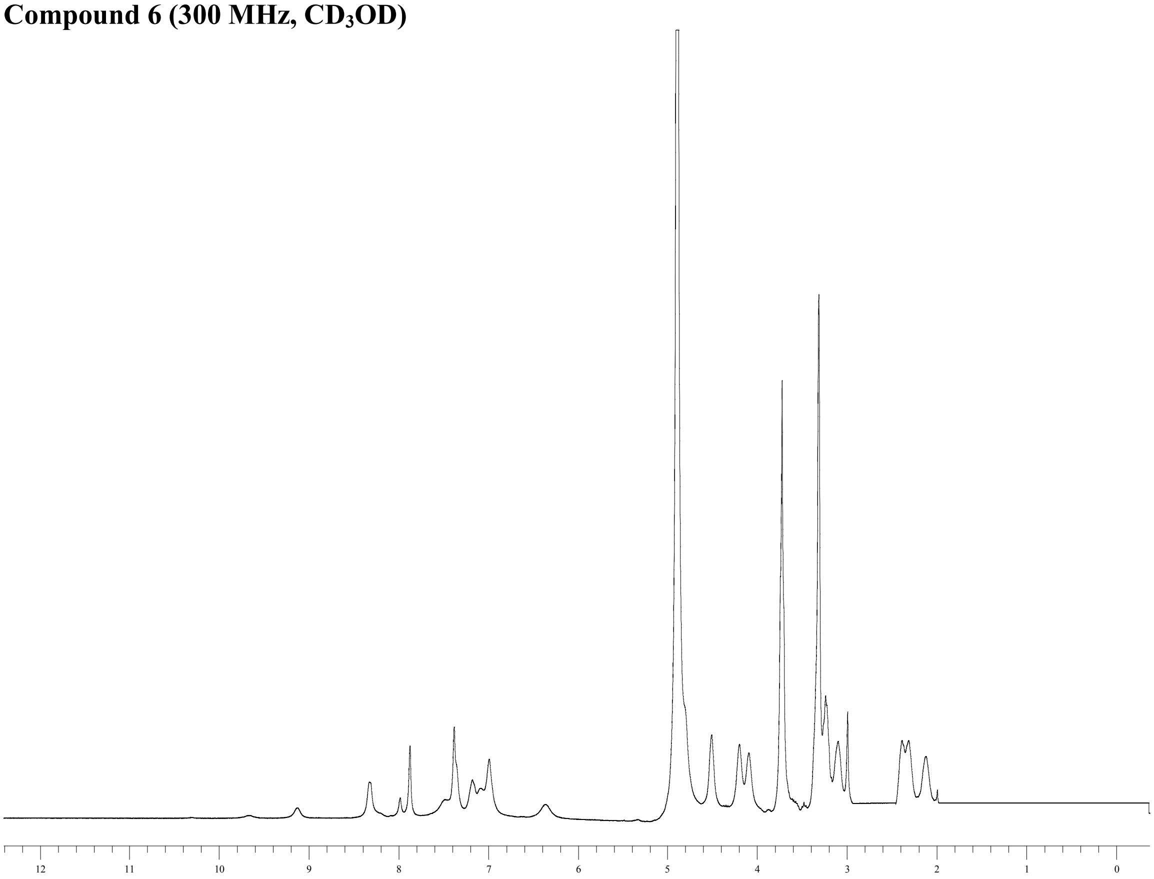

Compound 6 (HPLC chromatogram)

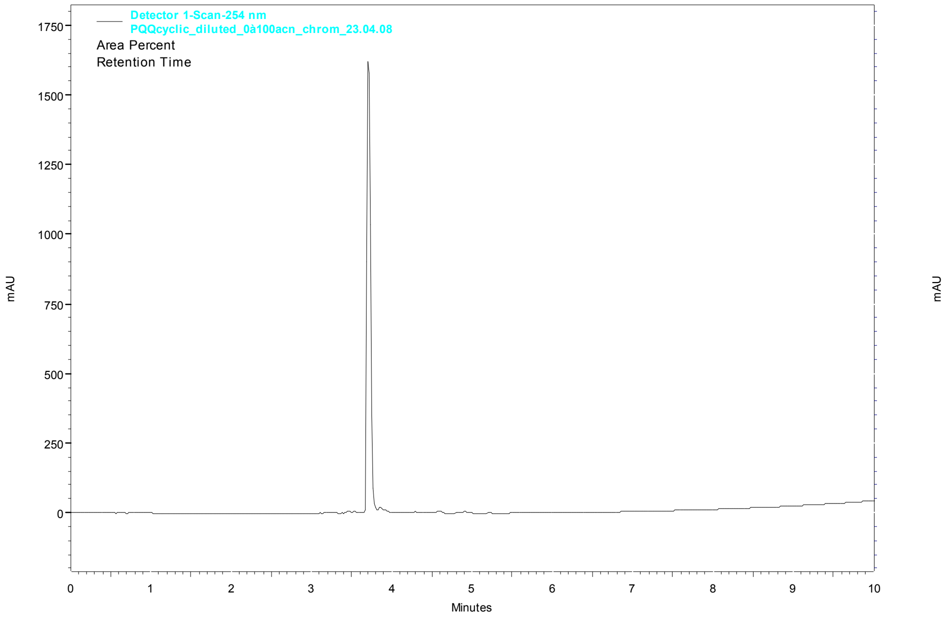

Compound 18 (300 MHz, $CDCl_3$)

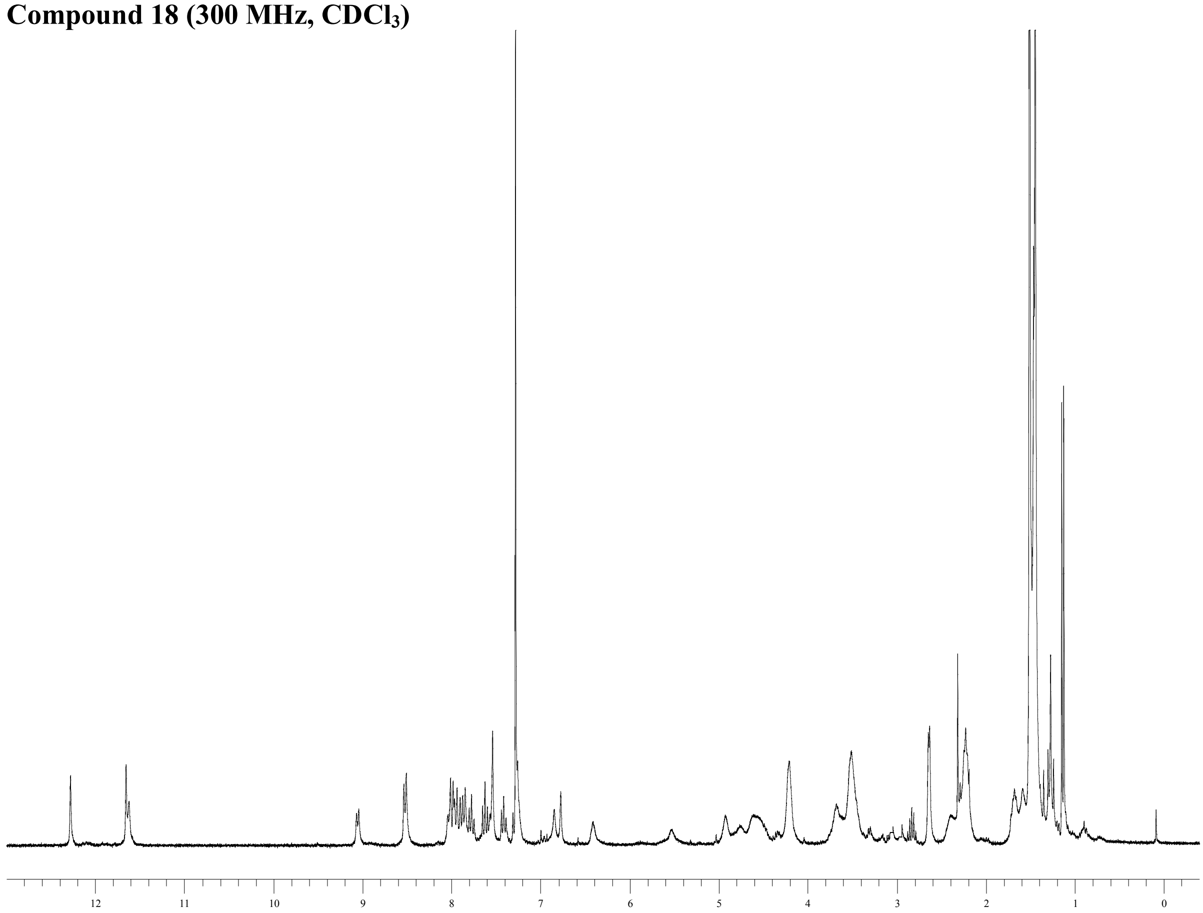

Compound 8 (300 MHz, CD_3OD)

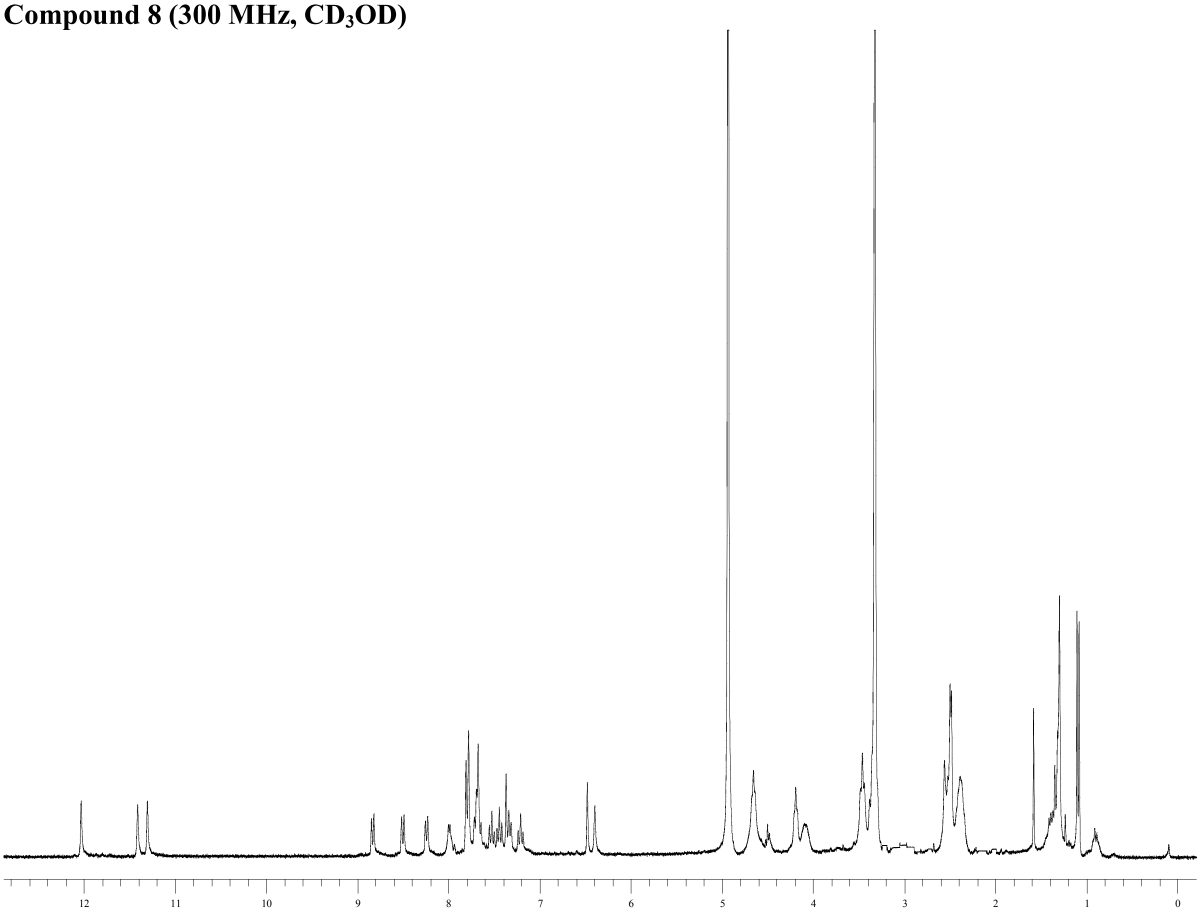

Compound 7 (300 MHz, CD_3OD)

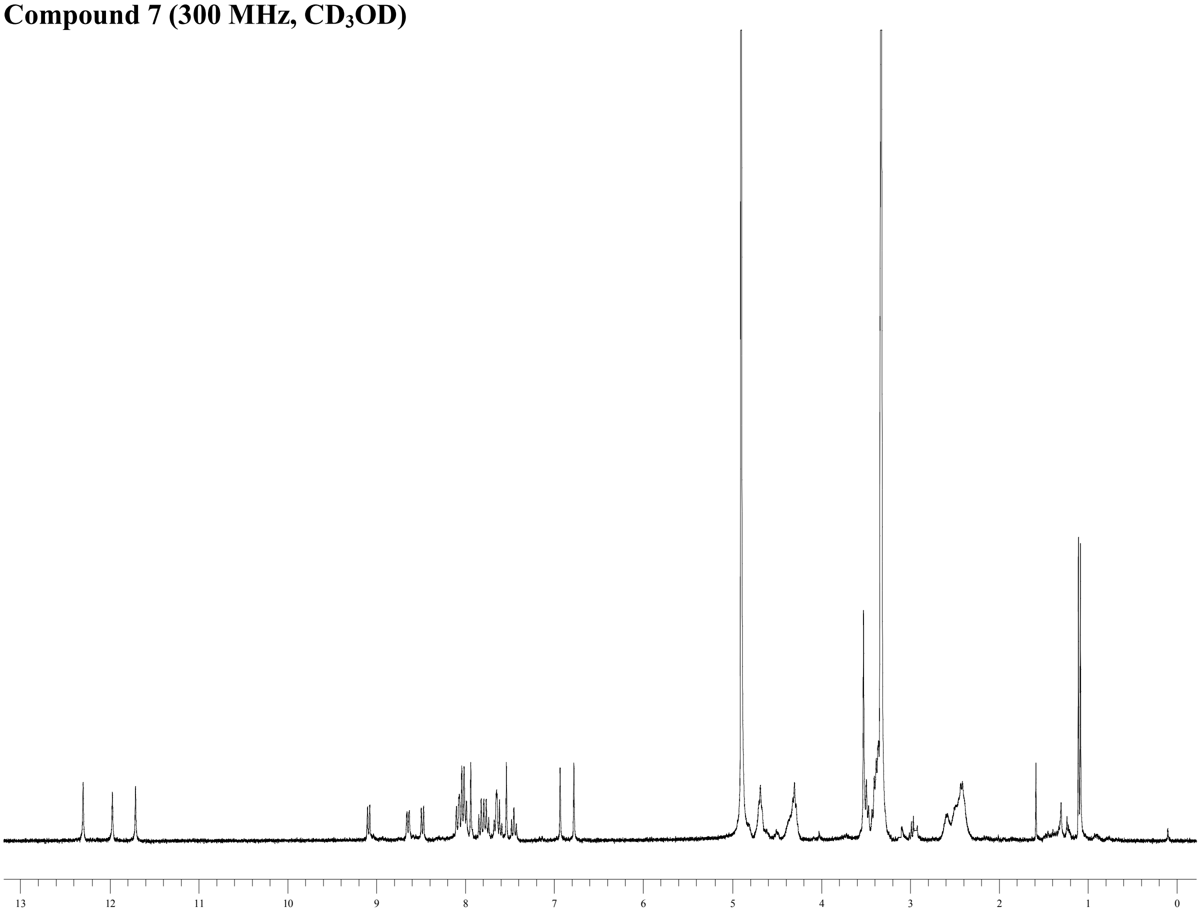

Compound 7 (HPLC Chromatogram)

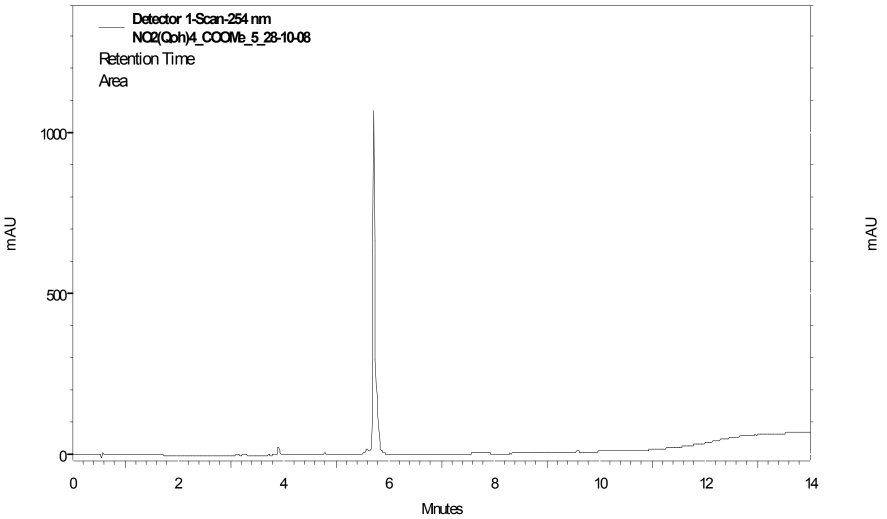

Compound 9 (300 MHz, CD_3OD)

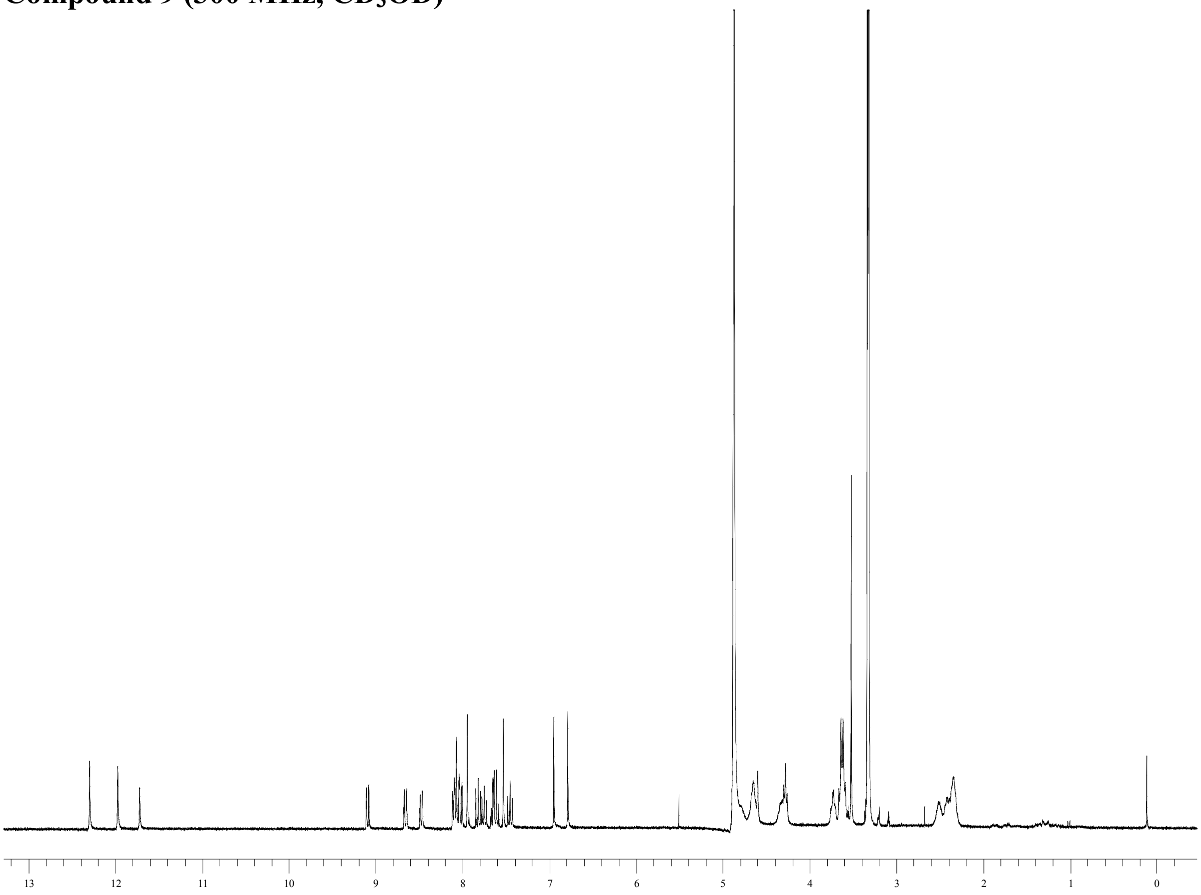

Compound 9 (HPLC Chromatogram)

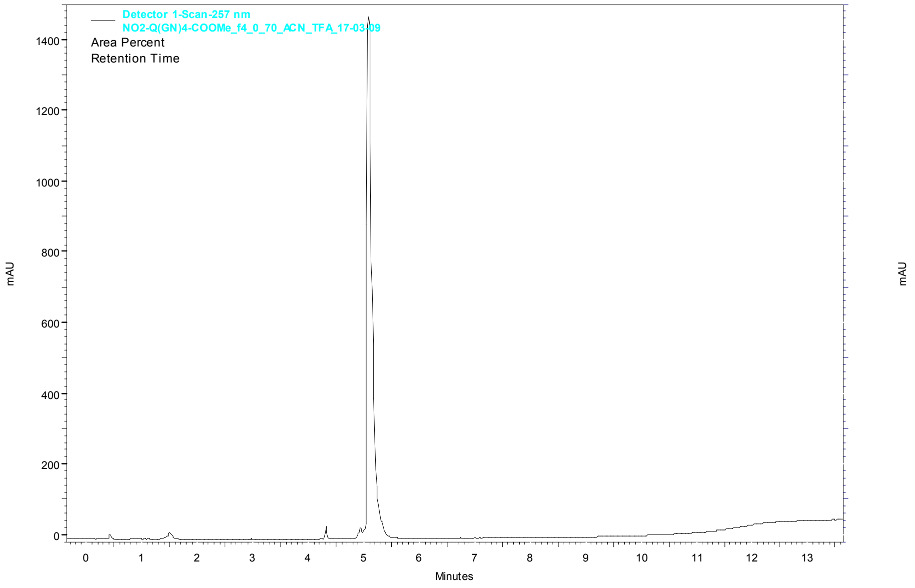

Compound 10 (300 MHz, CD_3OD)

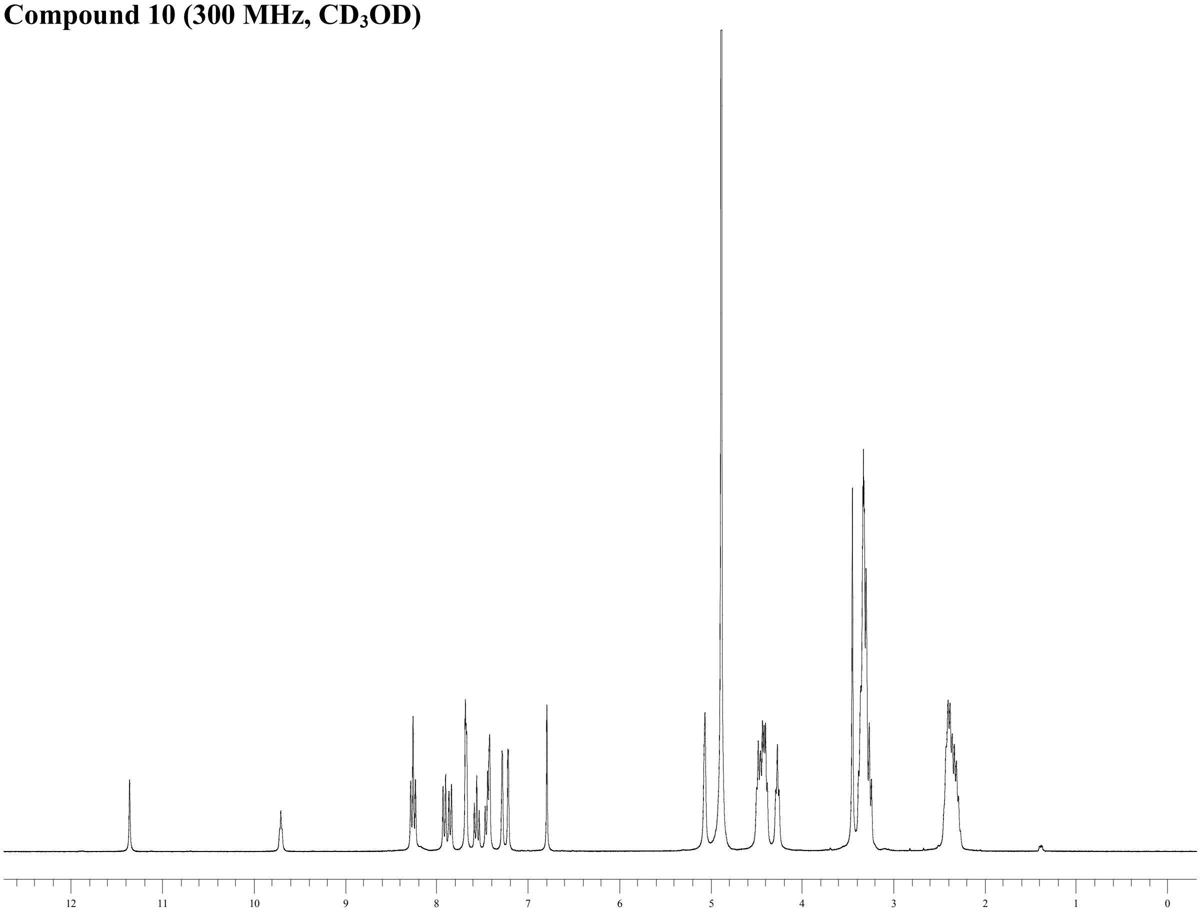

Compound 10 (HPLC Chromatogram)

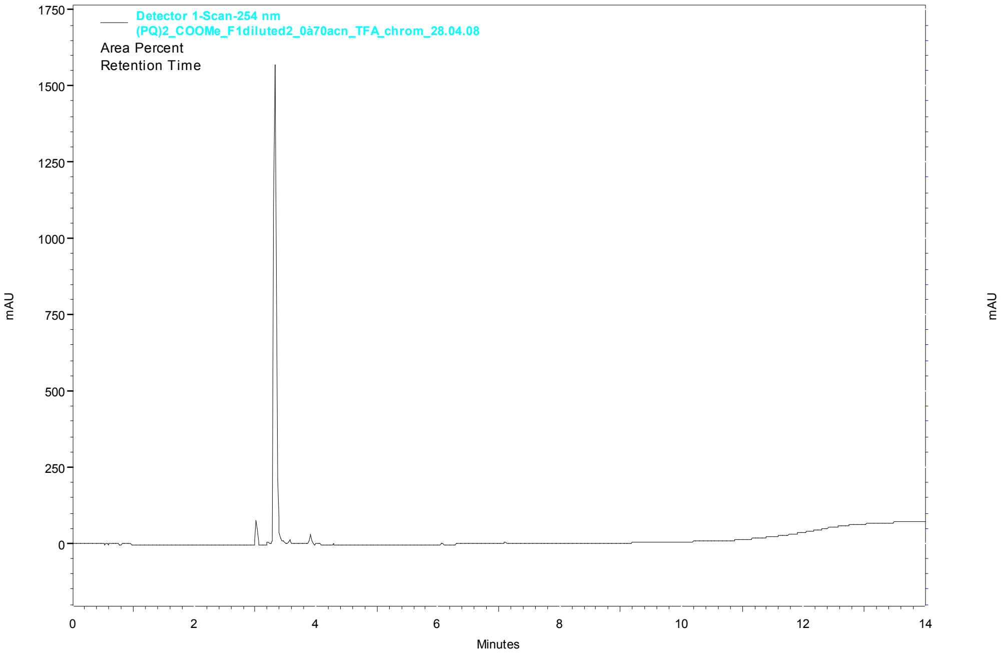

Compound 22a, 22c, 23a & 24a from top to bottom, respectively (300 MHz, $CDCl_3$)

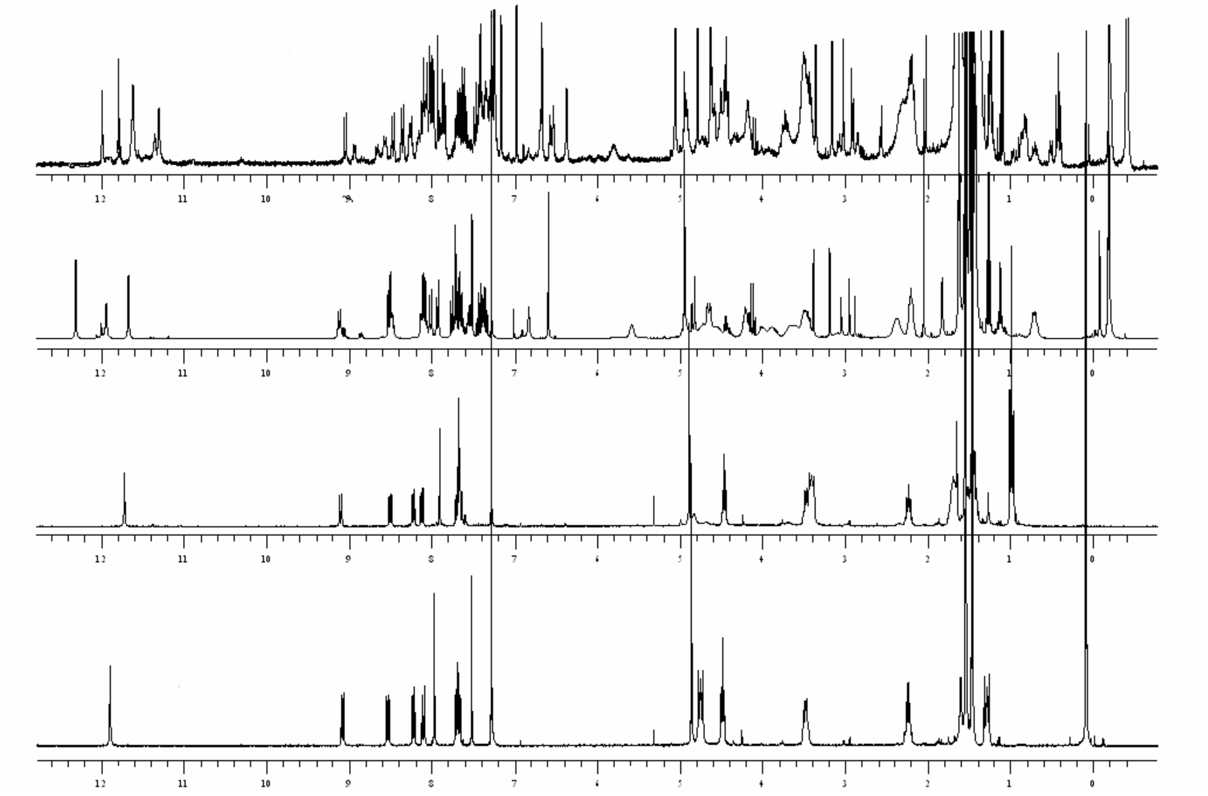

Compound 25a (300 MHz, DMSO-d$_6$)

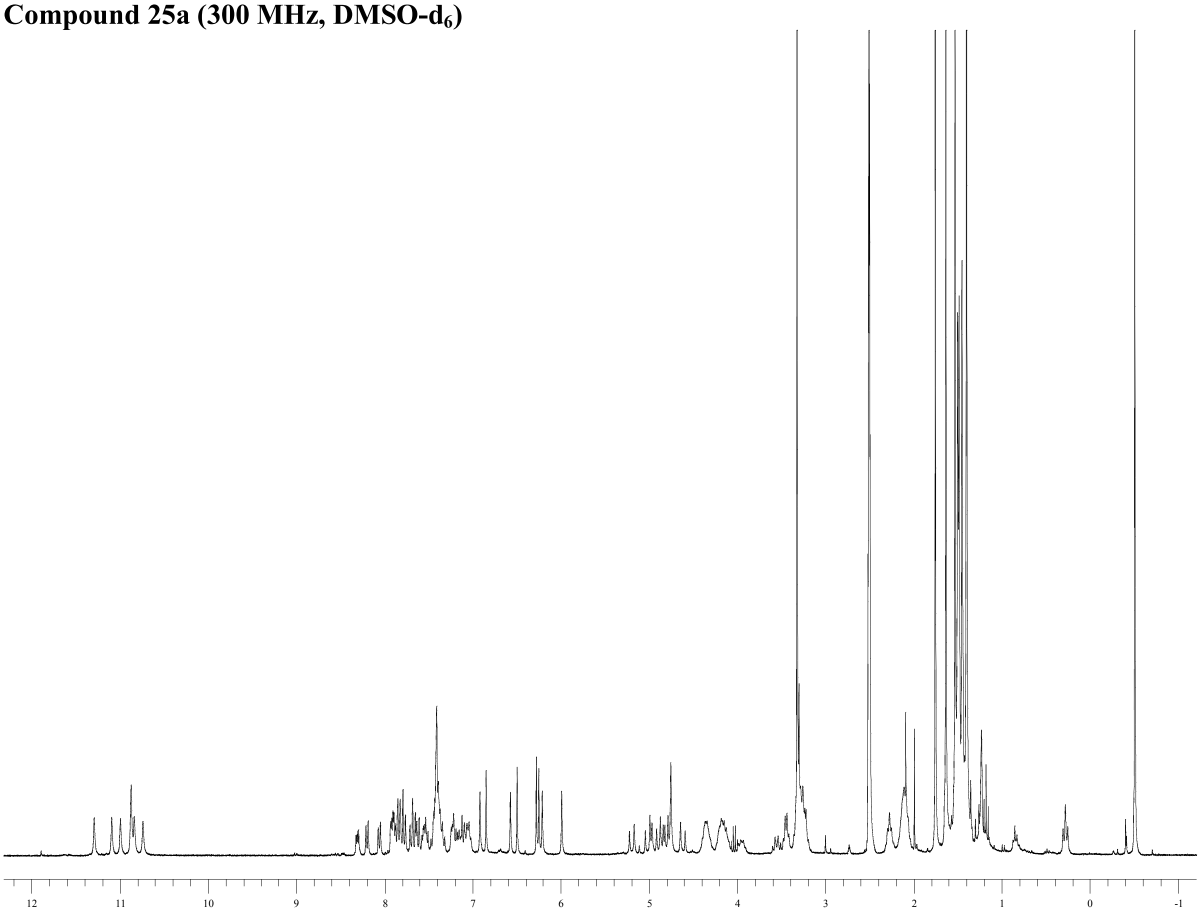

Compound 25b (300 MHz, DMSO-d$_6$)

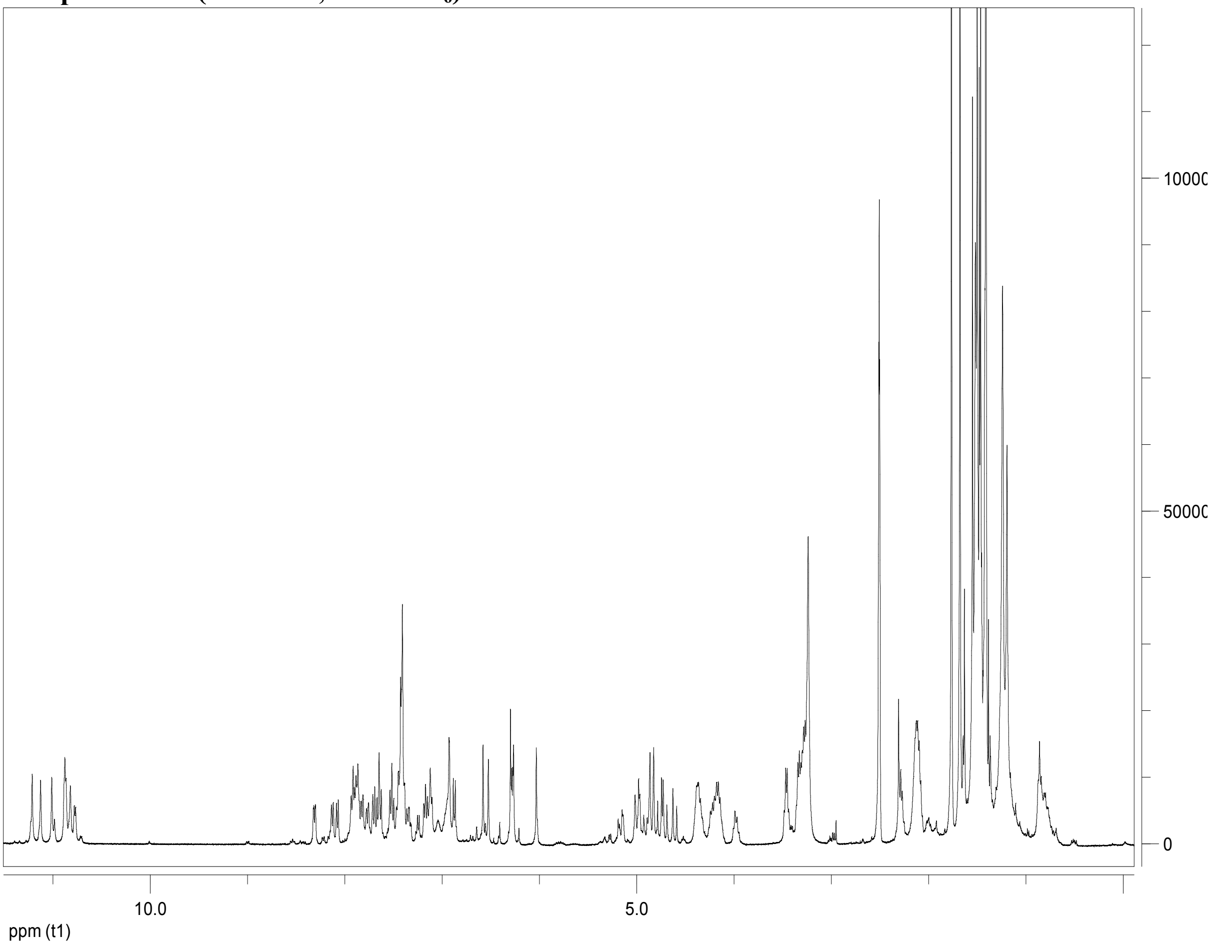

Compound 11 (300 MHz, CD_3OD/DMSO-d_6, 1:1 vol/vol)

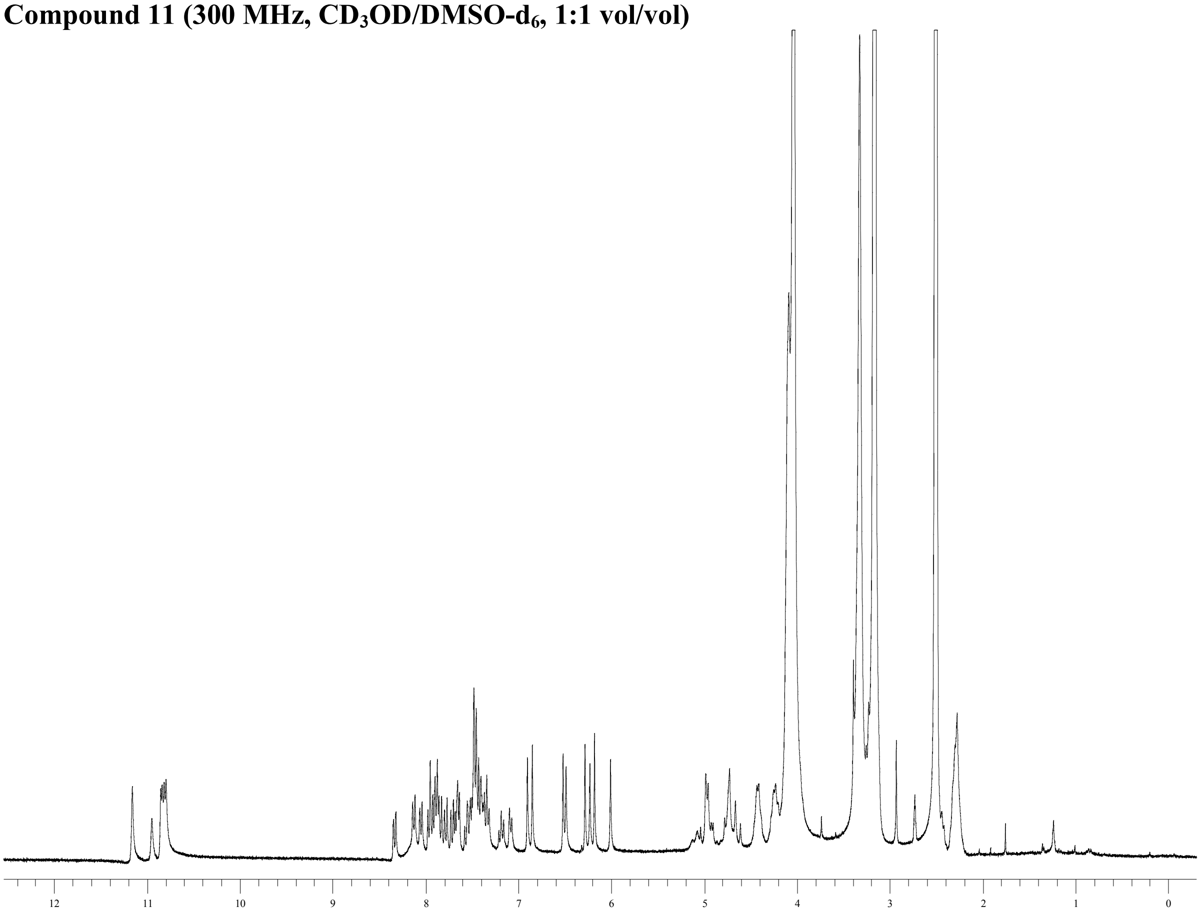

Compound 11 (HPLC Chromatogram)

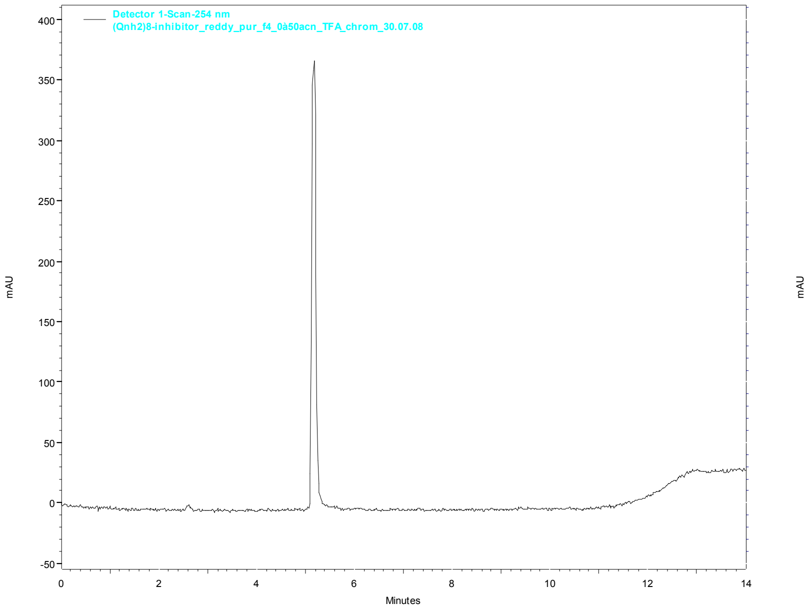

Compound 12 (300 MHz, DMSO-d_6)

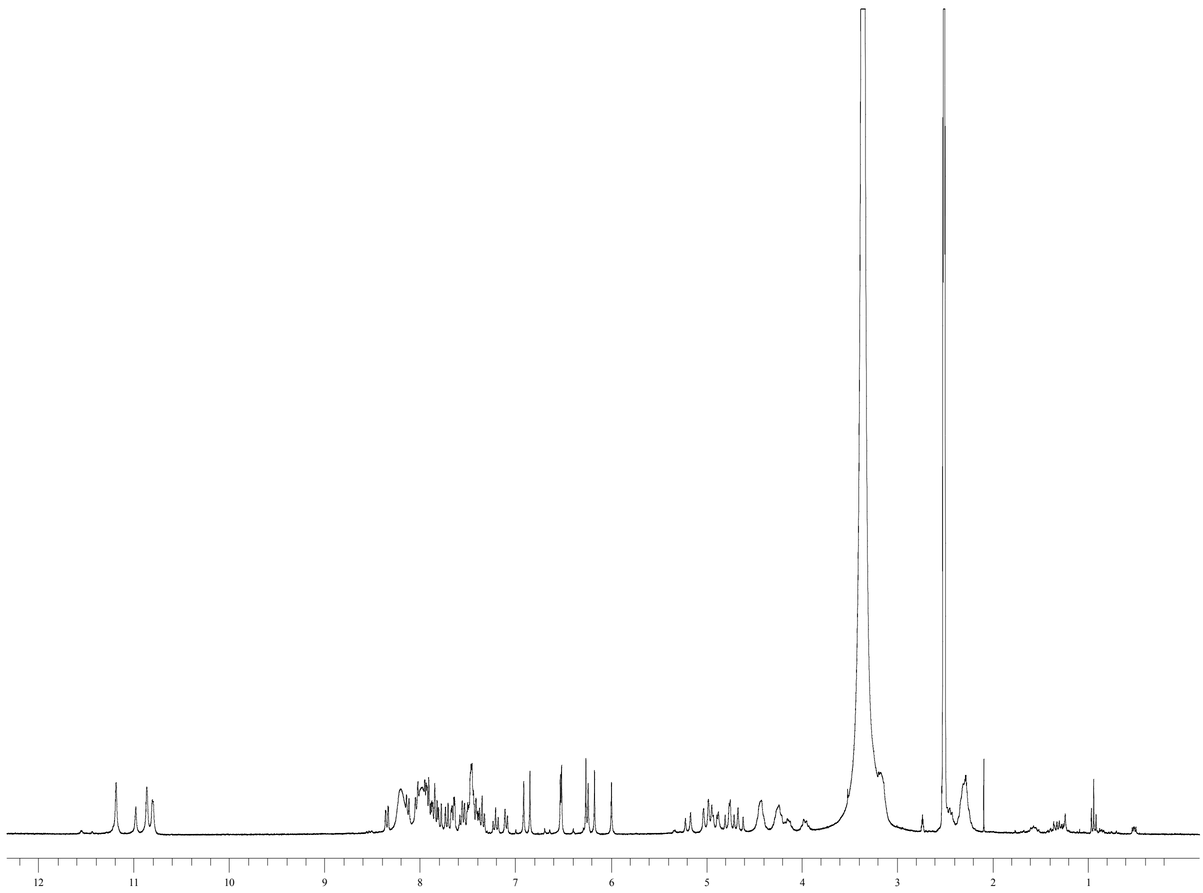

Compound 12 (HPLC Chromatogram)

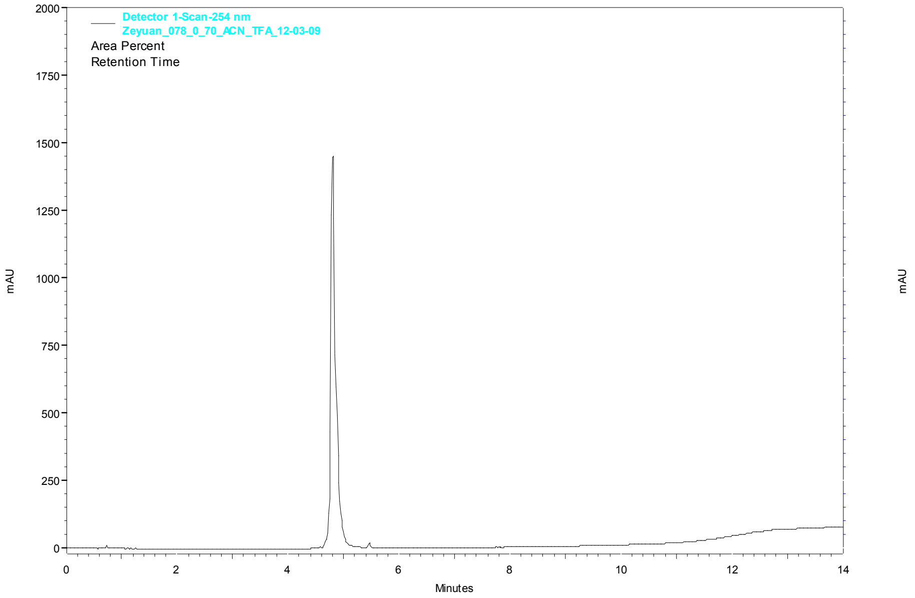

Supplement: Supplementary file 1 [file cbic0015-2563-sd1.pdf]
